# Towards a novel model for studying the nutritional stage dynamics of the Colombian population by age and socioeconomic status

**DOI:** 10.1371/journal.pone.0191929

**Published:** 2018-02-08

**Authors:** Jose D. Meisel, Olga L. Sarmiento, Camilo Olaya, Pablo D. Lemoine, Juan A. Valdivia, Roberto Zarama

**Affiliations:** 1 Department of Industrial Engineering, Faculty of Engineering, Universidad de los Andes, Bogotá, Colombia; 2 Facultad de Ingeniería, Universidad de Ibagué, Ibagué, Colombia; 3 CeiBA Complex Systems Research Center, Bogotá, Colombia; 4 Department of Public Health, School of Medicine, Universidad de los Andes, Bogotá, Colombia; 5 Centro Nacional de Consultoría, Bogotá, Colombia; 6 Departamento de Física, Facultad de Ciencias, Universidad de Chile, Santiago, Chile; 7 Centro para el Desarrollo de la Nanociencia y la Nanotecnología, CEDENNA, Santiago, Chile; California State Polytechnic University Pomona, UNITED STATES

## Abstract

Low-and middle-income countries (LMICs) are experiencing a nutritional transition in which the burden of obesity tends to shift towards the lower-socioeconomic status (SES) group. We propose a system dynamics (SD) model for assessing the nutritional stage dynamics of the Colombian urban population by age and SES projected to 2030. This SD model captures the ageing population according to body mass index (BMI) categories and SES. In this model, the transference rates (TRs) between BMI categories by age and SES are estimated using a heuristic based on data obtained from national surveys. The simulation results show that the Colombian population, particularly those aged 20 to 39 years with a lower SES, is moving towards the overweight and obese categories. The TRs for overweight and obese categories in the lower SES group (the mean TR from *not overweight* to *overweight* = 0.0215 (per year) and mean TR from *overweight* to *obese* = 0.0098 (per year)) are increasing more rapidly than the those in the middle (the mean TR from *not overweight* to *overweight* = 0.0162 (per year) and mean TR from *overweight* to *obese* = 0.0065 (per year)) and higher SES groups (the mean TR from *not overweight* to *overweight* = 0.0166 and mean TR from *overweight* to *obese* = 0.0054 (per year)). Additionally, from 2005 to 2010, individuals aged 20 to 39 years had the highest TRs towards the overweight and obese categories (from 0.026 to 0.036 per year and from 0.0064 to 0.012 per year, respectively). The TRs also indicated that children aged 0 to 14 years are moving from the obese to overweight and from the overweight to not overweight categories. These TRs show that the Colombian population is experiencing an SES-related nutritional transition that is affecting the lower SES population. The proposed model could be implemented to assess the nutritional transitions experienced in other LMICs.

## Introduction

Obesity is a major public health problem worldwide [1,2]. In fact, according to a recent policy report in 2010, a high body mass index (BMI) was the sixth leading risk factor for premature death and disability [3]. Similarly, the global mortality trends projected by the World Health Organization (WHO) predict that an increase in the total death rate by 2030 primarily due to non-communicable diseases (NCDs), particularly cancers and cardiovascular diseases and predominantly in low- and middle-income countries (LMICs) [4]. LMICs are currently experiencing a nutritional transition in which the burden of obesity is shifting towards those with a lower socio-economic status (SES) [5–7]. Moreover, in contrast to pre-1989 studies, which positively linked obesity with a high SES in LMICs but with a low SES in high-income countries (HICs) [8], recent research suggests that obesity in LMICs is not solely a disease of higher SES groups. In fact, obesity, particularly in women, tends to transition towards the lower-SES population as a country’s gross national product (GNP) increases [5,6].

This obesity shift is currently observable in Latin America [5,9], particularly in Colombia, where the prevalence of obesity among women of childbearing age in the lowest wealth index (WI) quintile increased by 27.66% from 2005 to 2010 compared to only a marginal increase of 4.24% in the highest WI quintile [10].

One promising framework for capturing this transition is a complex systems approach which investigates emergent phenomena over time by considering feedback loops and focusing on multi-level dynamic interactions among and between diverse actors [11,12]. The obesity problem inherently involves not only a diversity of actors (e.g., individuals and families of different SESs, industries, government agencies, and health-care providers) with different motivations (e.g., the transnational food industry is seeking expansion in new markets offering ultra-processed products [13,14], whereas government health care agencies seek to control the contents and requirements of trans fats in all foods) interacting to produce a range of outcomes (e.g., changes in living environments, diets and lifestyles) [12], but also different levels of scale (e.g., genetic, individuals, family, communities, regions, and countries) [12] and a set of interrelated factors (e.g., food production and consumption and physical activity (PA)) [15] and feedback mechanisms (e.g., the obesity system map) [15], all of which may concurrently influence the development of obesity and success, or failure, of prevention efforts [16]. Most importantly, complex systems analysis also addresses the unintended effects of system interventions and the long delays between causes and effects [17].

However, despite the myriad of suggestions stemming from statistical approaches that studied this nutritional transition [5,6,18], the available research evaluating the SES-related obesity dynamic as a complex system is limited [19]. In addition, the previous studies that have investigated the dynamics of obesity and its associated features tend to adopt one of the following three frameworks: (i) system dynamics (SD) models, which have been employed to study body-weight regulation [20–23] or to understand obesity dynamics at the population-level [24,19,25–28]; (ii) network analyses (NA), which have been conducted to study the spread of obesity [29] or obesity-related behaviours in friendship networks [30]; and (iii) agent-based models (ABM), which have been implemented to explore the possible role of economic segregation in generating income disparities in relation to diet [31] or to assess the effect of the Bus Rapid Transit system on walking [32].

At the population-level, which is the context of this study, Homer et al. [24] proposed an SD simulation model to study changes in obesity trends in the entire U.S. population. The model used four BMI categories in conjunction with the annual ageing of population cohorts categorized by age and sex. Dangerfield and Zainal Abidin [19] developed a SD model to study dietary intake and physical activity expenditure as well as their impact on changes in body weight and BMI among English children (2 to 15 years of age). Additionally, Rahmandad and Sabounchi [25] developed a multi-level SD model to study the dynamics of obesity in the United States over time. The model captures energy balance and weight changes over the course of individuals’ lives and aggregates these to model population-level trends. Similarly, Fallah-Fini et al. [26] developed a population-level SD model that estimates the energy imbalance gap in the adult US population, by gender-race/ethnicity and BMI categories. More recently, Zainal Abidin et al. [28] proposed an SD model that considers the interrelations among food intake, energy expenditure, physical measurement, and BMI in one complex human weight regulation system to simulate the effect of changes in the eating behaviour of British children on weight and obesity, by gender and into three age groups. However, to the best of our knowledge, only a few studies have applied a systemic approach to examine SES-related obesity dynamics. This study therefore investigates the nutritional stage dynamics within the urban population of Colombia stratified by both age and SES using a SD model. In particular, we propose a model that can be used: (1) to estimate the transference rates (TRs) between BMI categories by age and SES, (2) to identify the population subgroups towards which intervention efforts should be targeted, and 3) to monitor the potential effect of public health policy interventions aimed at preventing overweight and obesity across age and SES categories.

## Methods

### Model overview

To analyse nutritional stage dynamics, we first proposed a population-level SD model. We then calibrated the model and proposed a heuristic, which was based on previously published research [33], to estimate the TRs between BMI categories by age using data obtained from “*Encuesta Nacional de Demografía y Salud”* [ENDS] of 2005 and 2010 for urban individuals aged 0 to 64 years. Finally, we estimated the TRs between BMI categories by both SES and age and applied the SD model to the different SES groups. We only focused on the urban population because the highest SES population comprised less than 0.05% of the total population in rural areas [10].

### Data

The main data source used to inform the models was ENDS [10,34]. Our analysis is based on cross-sectional data from obtained from the ENDS of 2005 and 2010; a survey that included 27,794 and 36,412 urban households, respectively. The ENDS used a multistage population-based sampling design stratified by cluster (household segment) [10,34]. The study sample for 2005 comprised 8,515 children younger than five years, 32,009 children and adolescents aged 5–17 years, and 48,056 adults aged 18–64 years for whom objective anthropometric measurement data were available. The corresponding numbers for the 2010 sample were 11,368, 32,524, and 64,425, respectively. The questionnaire was administered in the home by interviewers equipped with computer-assisted personal interview technology. All the protocols were reviewed and approved by the Profamilia Institutional Review Board on Research Involving Human Participants.

Additional data were used to estimate the TRs stratified by BMI category and to simulate estimated fractions of the BMI categories over time for each age and SES group. In particular, the prevalence rates and fractions of births by BMI, age, and SES categories were obtained from data from the ENDS of 2005 and 2010 [35,36]; the population sizes and mortality rates, both stratified by age, were obtained from the Colombian National Department of Statistics (DANE) [37,38]; and the fertility rates from 2005 to 2014 were obtained from the World Data Bank [39]. The fertility rates from 2015 to 2030 were forecasted using the Holt-Winters no seasonal method in EViews 5 (Quantitative Micro Software, LLC). All the data used in the model were obtained from public sources and are fully available in S2 Appendix. Table 1 describes the data sources used in the model.

**Table 1:** Data sources used in the model^†^.

*Item*	*Data Source*
***Prevalence rates by BMI category*, *age*, *and WI***	
BMI for age and sex *z*-score for children and adolescents aged 0 to 17 years by WI	Colombia: Standard DHS, 2005 Dataset [35] Colombia: Standard DHS, 2010 Dataset [36]
BMI category prevalence by age and WI for adults	
***Population composition***	
Population size by age	DANE. Estimations and population projections for 1985–2020 [38]
Mortality rates by age group	DANE. Vital Statistics (2005) [37]
Fertility rate	World Data Bank. World Development Indicators (1960–2014) [39]. Projections for 2015–2030. Forecast series using the Holt-Winters no seasonal method in EViews 5 (Quantitative Micro Software, LLC)
Fraction of births by BMI category	Colombia: Standard DHS, 2005 Dataset [35]
***Classification of individuals by BMI categories***	
Height-for-age z-score and BMI for age and sex z-score cutoff points for children and adolescents aged 0 to 17 years: Not overweight: BMI for age and sex z-score ≤1 standard deviation. Overweight: BMI for age and sex z-score >1 standard deviation and ≤ 2 standard deviations. Obese: BMI for age and sex z-scores >2 standard deviations.	WHO child growth standards [40] WHO child growth references [41]
BMI category cut-points for adults aged 18 to 64 years: Not overweight: BMI <25 kg/m^2^ Overweight: BMI ≥25 and <30 kg/m^2^ Obese: BMI ≥30 kg/m^2^	WHO cutoff points [42]

†DHS = Demographic and Health Surveys; DANE = Colombian National Department of Statistics (for the Spanish acronym); WHO = World Health Organization; BMI = body mass index; WI = wealth index.

### Model of nutritional stage dynamics

The proposed population-level SD model includes ageing chains for three BMI categories (*not overweight*, *overweight*, and *obese*); thus, the population aged 0–59 years was divided into groups of 5 years. Because the actors themselves are heterogeneous–that is, the actors in each cohort according to the BMI category and age group show differences in physical activity (PA) level, dietary patterns, and BMI prevalence [10,34]–we sub-divided each age group into the BMI categories. The population ageing structure also included births and deaths for each cohort, with mortality rates differed among the age groups [33,43]. Given the purpose of the study and aims defined for the proposed SD model, we assumed the same mortality rate for each BMI category in each age group and that the net migration was zero. We made this assumption because currently there are no data for Colombia regarding mortality rates or net migration patterns by BMI category. Another reason we assumed that the net migration was zero, was because the proportion of individuals who migrate from Colombia is less than 0.5% per year [44].

We classified individuals into the three BMI categories based on the following WHO criteria: the 2006 WHO child growth standards for children under 5 [40], the 2006 WHO child growth references for children aged 5 to 17 years [41], and the WHO cut-off points for aged at least 18 years [42]. For children and adolescents aged 0–17 years, the WHO system defines *not overweight* as a BMI for age and sex *z*-score ≤1 standard deviation, *overweight* as a BMI for age and sex *z*-score >1 standard deviation and ≤ 2 standard deviations, and *obese* as a BMI for age and sex *z*-score >2 standard deviations. For adults, WHO defines *not overweight* as a BMI <25 kg/m^2^, *overweight* as a BMI ≥25 and <30 kg/m^2^, and *obese* as a BMI ≥30 kg/m^2^ (Table 1).

**Fig 1:**
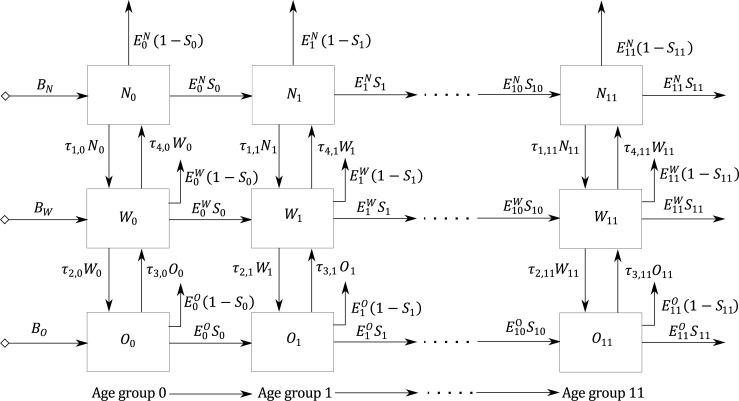
Overview of the SD model structure for the entire urban population.

The mathematical form of the model is shown below (Eqs (1–3) correspond to the net rate of change of the population in each BMI category for the age group of zero to four years. Eqs (4–6) correspond to the net rate of change of the population in each BMI category for the rest of the age groups):

$$\frac{dN_{0}(t)}{dt}=B_{N}(t)+{\tau_{4,0}W}_{0}(t)-{\tau_{1,0}N}_{0}(t)-E_{0}^{N}(t)(1-S_{0})-E_{0}^{N}(t)S_{0}$$ (1)

$$\frac{dW_{0}(t)}{dt}=B_{W}(t)+{\tau_{1,0}N}_{0}(t)+{\tau_{3,0}O}_{0}(t)-W_{0}(t)(\tau_{2,0}+\tau_{4,0})-E_{0}^{W}(t)(1-S_{0})-E_{0}^{W}(t)S_{0}$$ (2)

$$\frac{dO_{0}(t)}{dt}=B_{O}(t)+{\tau_{2,0}W}_{0}(t)-{\tau_{3,0}O}_{0}(t)-E_{0}^{O}(t)(1-S_{0})-E_{0}^{O}(t)S_{0}$$ (3)

$$\frac{dN_{i}(t)}{dt}={E_{i-1}^{N}(t)S_{i-1}+\tau_{4,i}W}_{i}(t)-{\tau_{1,i}N}_{i}(t)-E_{i}^{N}(t)(1-S_{i})-E_{i}^{N}(t)S_{i},\;for\;i\;\in (1,\ldots ,n-1)$$ (4)

$$\frac{dW_{i}(t)}{dt}=E_{i-1}^{W}(t)S_{i-1}+{\tau_{1,i}N}_{i}(t)+{\tau_{3i}O}_{i}(t)-W_{i}(t)(\tau_{2,i}+\tau_{4,i})-E_{i}^{W}(t)(1-S_{i})$$

$$-E_{i}^{W}{(t)S}_{i},\;for\;i\;\in (1,\ldots ,n-1)$$ (5)

$$\frac{dO_{i}(t)}{dt}=E_{i-1}^{O}(t)S_{i-1}+{\tau_{2,i}W}_{i}(t)-{\tau_{3,i}O}_{i}(t)-E_{i}^{O}(t)(1-S_{i})-E_{i}^{O}(t)S_{i},\;for\;i\;\in (1,\ldots ,n-1)$$ (6)

where *i* ∈ (0,….,11) represents the age groups, in intervals of 5 years, of the simulated population (0–4, 5–9,…,55–59); *n* corresponds to the total number of age groups (12); *N*_*i*_*(t)*, *W*_*i*_*(t)*, and *O*_*i*_*(t)* are the populations of *not-overweight*, *overweight*, and *obese* individuals in the age group *i*, respectively, at time *t* (unit: people); *B*_*N*_*(t)*, *B*_*W*_*(t)*, and *B*_*O*_*(t)* are the births for each BMI category of the first age group at time *t* (unit: people per year); and *E*_*i*_^*N*^*(t)*, *E*_*i*_^*W*^*(t)*, and *E*_*i*_^*O*^*(t)* are the exit rates of individuals per year for each age group *i* and BMI category at time *t* (unit: people per year). An exit rate is the total number of individuals per year that leave each age group by BMI category. There are two groups of individuals to whom the exit rate is applied: those who mature into the next age group (e.g., *E*_*i*_^*O*^*(t)S*_*i*_) and those who die (e.g., *E*_*i*_^*O*^*(t)(1-S*_*i*_)); *S*_*i*_ is the survival fraction per year for each age group *i* (unit: % per year). The parameters *τ*_*1*,*i*_ and *τ*_*2*,*i*_ are the TRs that correspond to the fraction of individuals per year from the *not overweight* and the *overweight* categories that become *overweight* and *obese* for each age group *i*, respectively (unit: % per year). The parameters *τ*_*3*,*i*_ and *τ*_*4*,*i*_ are the TRs of individuals from the *obese* to the *overweight* category and from the *overweight* to the *not overweight* category, respectively, from each age group *i* (unit: % per year) (see the flow diagram in Fig 1 for a global view of the population aging structure). The case of *i* = 0, which corresponds to ages zero to four years, is shown explicitly within the figure because of its slight difference from the other age groups. When people reach the age of 60 they exit the system. The reason people in the 60 to 64 age group are not represented within the SD model is because the data that would allow for a reliable estimate of the TRs for this age group–namely, prevalence rates by BMI category for the age group 65 to 69 in 2010—is unavailable.

The number of births per year for each BMI category was determined using the following equations:

$$B_{N}(t)=\mu \theta_{N}(\frac{f(t)}{\left(Y_{F}-Y_{I}+1\right)})\sum_{i=Y_{I}}^{Y_{F}}\left(N_{i}(t)+W_{i}(t)+O_{i}(t)\right)$$ (7)

$$B_{W}(t)=\mu \theta_{W}(\frac{f(t)}{\left(Y_{F}-Y_{I}+1\right)})\sum_{i=Y_{I}}^{Y_{F}}\left(N_{i}(t)+W_{i}(t)+O_{i}(t)\right)$$ (8)

$$B_{O}(t)=\mu \theta_{O}\left(\frac{f(t)}{\left(Y_{F}-Y_{I}+1\right)}\right)\sum_{i=Y_{I}}^{Y_{F}}\left(N_{i}(t)+W_{i}(t)+O_{i}(t)\right)$$ (9)

where *μ* is the total fraction of women in the population aged 15 to 49 years (we used the fraction of urban women in 2005 according to the ENDS survey, *μ = 0*.*523*, unit: % per year); *μ∑*_*i = YI*_^*YF*^*((N*_*i*_*(t) + W*_*i*_*(t) + O*_*i*_*(t))* is the female population of childbearing age at time *t* (unit: women); *f* is the total number of children born from each woman during the childbearing years (fertility rate) at time *t* (births for each woman, unit: child per woman); *θ*_*N*_, *θ*_*W*_, and *θ*_*O*_ are the fractions of births by each BMI category (unit: % per year) (assumed to be the prevalences by BMI category of children aged 0 to 2 months); and Y_I_ and Y_F_ (unit: year) are the first and last childbearing years considered, which we assumed to be 15 to 49, respectively.

The exit rates per year by each age group and BMI category were modelled using the following equations:

$$E_{0}^{N}(t)=\left(\frac{B_{N}(t)+{\tau_{4,0}W}_{0}(t)-{\tau_{1,0}N}_{0}(t)}{Y}\right)$$ (10)

$$E_{0}^{W}(t)=\left(\frac{B_{W}(t)+{\tau_{1,0}N}_{0}(t)+{\tau_{3,0}O}_{0}(t)-W_{0}(t)(\tau_{2,0}+\tau_{4,0})}{Y}\right)$$ (11)

$$E_{0}^{O}(t)=\left(\frac{B_{O}(t)+{\tau_{2,0}W}_{0}(t)-{\tau_{3,0}O}_{0}(t)}{Y}\right)$$ (12)

$$E_{i}^{N}(t)=\left(\frac{E_{i-1}^{N}{(t)S}_{i-1}+{\tau_{4,i}W}_{i}(t)-{\tau_{1,i}N}_{i}(t)}{Y}\right),\;for\;i\;\in (1,\ldots ,n-1)$$ (13)

$$E_{i}^{W}(t)=\left(\frac{E_{i-1}^{W}(t)S_{i-1}+{\tau_{1,i}N}_{i}(t)+{\tau_{3,i}O}_{i}(t)-W_{i}(t)(\tau_{2,i}+\tau_{4,i})}{Y}\right),\;for\;i\;\in (1,\ldots ,n-1)$$ (14)

$$E_{i}^{O}(t)=\left(\frac{E_{i-1}^{O}(t)S_{i-1}+{\tau_{2,i}W}_{i}(t)-{\tau_{3,i}O}_{i}(t)}{Y}\right),\;for\;i\;\in (1,\ldots ,n-1)$$ (15)

where *Y = 5 years*, corresponding to the average time individuals spend in a given age group before maturing into the next age group–this being consistent with the five year intervals within the model. The Eqs (10–12) correspond to the exit rates per year by BMI category for the age group zero to four years. Finally, the survival fraction for each age group is determined by:

$$S_{i}=\exp\left(R_{i}Y\right),\;for\;i\;\in (0,\ldots ,n-1)$$ (16)

In this formulation, *R*_*i*_ is the mortality rate per year for each age group *i* (unit: % per year). We assumed the same mortality rates for each BMI category in each age group *i*, but differed across age groups.

The data processing was conducted using SAS 9.3 (SAS Institute Inc.) and Mathematica 9.0.1 (Wolfram Research, Inc.), and all simulations were run on iThink 9.0.2 (ISEE Systems, Inc.).

### Model calibration and parameter estimation

We developed five tests to assess the suitability of the proposed SD model in achieving its intended purpose [43]: integration error, parameter assessment, extreme conditions, behaviour reproduction, and sensitivity analysis. The results of these tests showed that the model was robust and that the behaviour pattern of the model were consistent with the expected results for the estimated prevalence rates for each BMI category. An explanation of each model test used to assess the SD model, as well as their results, is available in the S1 Appendix.

We developed a parameter assessment to determine whether the estimated TRs were consistent with relevant descriptive and numerical data. We proposed a heuristic for estimating the TRs (parameters) between BMI categories by age using data from the ENDS of the 2005 and 2010 (historical data). We initialized the heuristic using the distribution of BMI categories from 2005 ENDS which provides information about BMI categories by age in Colombia. This survey however, only allows tracing the growth of individuals by BMI categories and ages 0 to 64 years for 2005 and 2010 (previous ENDS surveys only provide data on BMI categories for some subgroups of the population). For the purposes of this SD model, we assumed that the TRs are uniform within each five-year age group, but differed across age groups.

To calculate the TRs by BMI category and age group we employed the following heuristic. First, we estimate the prevalence rates by BMI category and age *j* in 2010 for each age group *i* using the following system of equations:

$$P_{i+1,j}^{\prime }=B_{i}.P_{i,j}$$ (17)

where

$$B_{i}=A_{i}.(A_{i}.(A_{i}.(A_{i}.A_{i})));$$

$$A_{i}=\left(\begin{array}{ccc} \alpha_{1,i}-\tau_{1,i} & \tau_{4i} & 0\\ \tau_{1,i} & \alpha_{2,i}-\tau_{2,i}-\tau_{4,i} & \tau_{3i}\\ 0 & \tau_{2,i} & \alpha_{3,i}-\tau_{3,i} \end{array}\right);P_{i+1,j}^{\prime }=\left(\begin{array}{c} p_{N10,i+1,j}^{\prime }\\ p_{W10,i+1,j}^{\prime }\\ p_{O10,i+1,j}^{\prime } \end{array}\right);P_{i,j}=\left(\begin{array}{c} P_{N05,i,j}\\ P_{W05,i,j}\\ P_{O05,i,j} \end{array}\right);$$

where *j* ∈ (1,….,5) is the corresponding year in each age group *i*; *i* ∈ (0,….,11) represents intervals of five years (0–4, 5–9,…,55–59, and 60–64); *P*_*N05*,*i*,*j*_, *P*_*W05*,*i*,*j*_, and *P*_*O05*,*i*,*j*_ are the prevalence rates by BMI category and age *j* in 2005 for age group *i*; *α*_*1*,*i*_, *α*_*2*,*i*_, and *α*_*3*,*i*_ are the retention rates for individuals by BMI category and age group *i*, corresponding to the fraction of individuals who remain in the same BMI category between 2005 and 2010; *τ*_*1*,*i*_, *τ*_*2*,*I*,_ *τ*_*3*,*i*_, and *τ*_*4*,*i*_ are the TRs that are used to run the proposed SD model; and *P’*_*N10*,*i+1*,*j*_, *P’*_*W10*,*i+1*,*j*_, and *P’*_*O10*,*i+1*,*j*_ are the estimated prevalence rates by BMI category and age *j* in 2010 for age group *i* five years later. For example, we supposed that the individuals who were 5 years of age in 2010 were the same individuals who were 0 years of age in 2005. The matrix *A*_*i*_ represents the equations used to calculate the estimated prevalence rates by BMI category in the year *t+1* for age group *i*, where *t* is 2005. To estimate the prevalence rates by BMI category and age group in the year 2010 (*t+5*), the heuristic considers changes in the prevalence rates by BMI category for each year as a function of the prevalence rates by BMI categories obtained from 2005 ENDS. This heuristic therefore, estimates the TRs aggregated over five years.

After estimating the prevalence rates by BMI category and age group in 2010, the heuristic calculates the TRs by BMI category and age group using a minimization process. This process involves minimizing the difference between prevalence rates by BMI categories and age groups reported in the 2010 ENDS and the estimated 2010 prevalence rates informed by data from the 2005 ENDS. Specifically, the TRs were estimated by solving the system of Eq (17) for each age group *i*, and minimizing the following equation using the FindMinimum function in Mathematica 9 [33]:

$${Min\;CD}_{i}=\sum_{j=1}^{5}\left[{\left(P_{N10,i+1,j}-p_{N10,i+1,j}^{\prime }\right)}^{2}+{\left(P_{W10,i+1,j}-p_{W10,i+1,j}^{\prime }\right)}^{2}+{\left(P_{O10,i+1,j}-p_{O10,i+1,j}^{\prime }\right)}^{2}\right],$$ (18)

with the restrictions

$$\alpha_{1,i}+\tau_{1,i}=1$$

$$\alpha_{2,i}+\tau_{2,i}+\tau_{4,i}=1$$

$$\alpha_{3,i}+\tau_{3,i}=1$$ (19)

$$0\leq \alpha_{k,i}\leq 1,(k=1,2,3)$$

$$0\leq \tau_{l,i}\leq 1,(l=1,2,3,4)$$

where *P*_*N10*,*i+1*,*j*_, *P*_*W10*,*i+1*,*j*_, and *P*_*O10*,*i+1*,*j*_ are the prevalence rates by BMI category and age *j* in 2010 for age group *i*. The FindMinimum function searches for a local minimum in a function including several variables and constraints. The results of the parameter assessment showed that the quadratic differences between the prevalence rates by BMI categories reported by the ENDS in 2010 and the estimated 2010 prevalence rates predicted by the system of Eq (17) were less than 5% for each age and SES group. These values indicates that the prevalence rates predicted by the heuristic closely replicate the prevalence rates observed in 2010 (historical data). The results of the parameter assessment are described in the S1 appendix, Table 2 and Table 3.

**Table 2:** Estimated transference rates between BMI categories by age group for the entire urban population ^†^.

Age group	*Not overweight* to *overweight* (*τ*_*1*_)	*Overweight* to *obese* (*τ*_*2*_)	*Obese* to *overweight* (*τ*_*3*_)	*Overweight* to *not overweight* (*τ*_*4*_)
0–4	3.7564E-03	1.9989E-03	3.2706E-08	3.3883E-02
5–9	5.0334E-03	2.3978E-07	4.3462E-07	1.6578E-08
10–14	8.6916E-04	4.8673E-03	2.2426E-02	5.8634E-13
15–19	1.8773E-02	5.4279E-03	4.2342E-10	2.6586E-10
20–24	3.3429E-02	6.3982E-03	9.1309E-08	3.2073E-08
25–29	3.5826E-02	1.2145E-02	9.3667E-09	5.1414E-09
30–34	2.7964E-02	7.9848E-03	4.3676E-08	2.6979E-08
35–39	2.6149E-02	8.3326E-03	1.4624E-07	1.1489E-07
40–44	2.4555E-02	7.5370E-03	3.3784E-08	1.3284E-08
45–49	1.3626E-02	7.9259E-03	3.9024E-08	4.0720E-08
50–54	1.1986E-02	6.6221E-03	3.7719E-08	3.3221E-08
55–59	7.0130E-03	2.0203E-05	9.4052E-04	1.3984E-05

^**†**^ Age specific values estimated using the proposed heuristic.

**Table 3:** Estimated transference rates between BMI categories by age and SES group^†^.

SES group	Age group	*Not overweight* to *overweight* (*τ*_*1*_)	*Overweight* to *obese* (*τ*_*2*_)	*Obese* to *overweight* (*τ*_*3*_)	*Overweight* to *not overweight* (*τ*_*4*_)
Lower	0–4	1.3101E-03	1.0178E-09	1.0385E-04	4.6690E-02
	5–9	5.4557E-03	9.4718E-03	3.9426E-05	1.3408E-06
	10–14	4.6920E-03	3.9616E-03	6.3557E-05	2.3025E-08
	15–19	2.1034E-02	1.2332E-02	2.1903E-07	7.4870E-09
	20–24	3.7871E-02	7.6258E-03	1.9824E-09	7.2788E-10
	25–29	3.8596E-02	1.5073E-02	5.6016E-09	7.7021E-09
	30–34	3.1630E-02	1.5140E-02	8.3486E-09	1.8000E-09
	35–39	3.3527E-02	1.2201E-02	2.7137E-08	3.1316E-08
	40–44	2.8545E-02	1.6784E-02	2.5729E-08	2.2934E-08
	45–49	1.8423E-02	1.2963E-02	5.5552E-07	4.4111E-07
	50–54	2.3425E-02	8.1058E-03	2.8806E-07	3.1162E-07
	55–59	1.4192E-02	4.1445E-03	1.1794E-06	1.7226E-07
Middle	0–4	2.7128E-03	3.2160E-07	5.8332E-06	3.6038E-02
	5–9	5.8460E-03	5.3030E-08	2.3327E-02	1.4725E-03
	10–14	3.6125E-11	3.6311E-03	4.1365E-02	8.0070E-03
	15–19	1.7817E-02	4.1455E-03	5.8434E-07	3.1814E-08
	20–24	3.0706E-02	7.7520E-03	2.0614E-10	1.4237E-09
	25–29	3.3597E-02	1.3152E-02	4.1736E-12	6.1903E-12
	30–34	2.2712E-02	9.8560E-03	1.0235E-08	4.0128E-09
	35–39	2.2688E-02	1.2577E-02	7.0527E-08	7.7958E-08
	40–44	2.5588E-02	6.6106E-03	1.5016E-08	4.8602E-08
	45–49	1.0972E-02	1.1705E-02	2.7996E-06	2.9301E-07
	50–54	1.0326E-02	8.9437E-03	2.3323E-07	8.4381E-08
	55–59	1.0859E-02	4.3522E-08	3.4101E-03	2.1121E-03
Higher	0–4	6.1520E-03	2.3632E-02	3.0024E-11	8.9323E-10
	5–9	3.7007E-03	1.8209E-03	5.9251E-03	2.8633E-08
	10–14	2.9523E-07	2.7947E-09	4.2175E-02	2.1652E-02
	15–19	1.7719E-02	3.4803E-03	4.0073E-08	1.0205E-08
	20–24	3.1893E-02	5.2272E-03	1.2582E-08	5.3230E-09
	25–29	3.5510E-02	9.0317E-03	1.8883E-09	1.0375E-09
	30–34	2.8969E-02	2.5534E-03	5.1140E-10	1.5520E-09
	35–39	2.3850E-02	4.1629E-03	9.7547E-10	8.0792E-09
	40–44	2.2747E-02	3.8605E-03	7.3569E-09	2.3592E-08
	45–49	1.5990E-02	4.7485E-03	8.3930E-08	8.9747E-07
	50–54	5.4516E-03	5.9228E-03	1.4811E-06	1.2808E-07
	55–59	7.0109E-03	8.2735E-08	2.6205E-03	1.9005E-03

^**†**^ Age- and SES-specific values estimated using the proposed heuristic.

### Model of nutritional stage dynamics by BMI and SES

**Fig 2:**
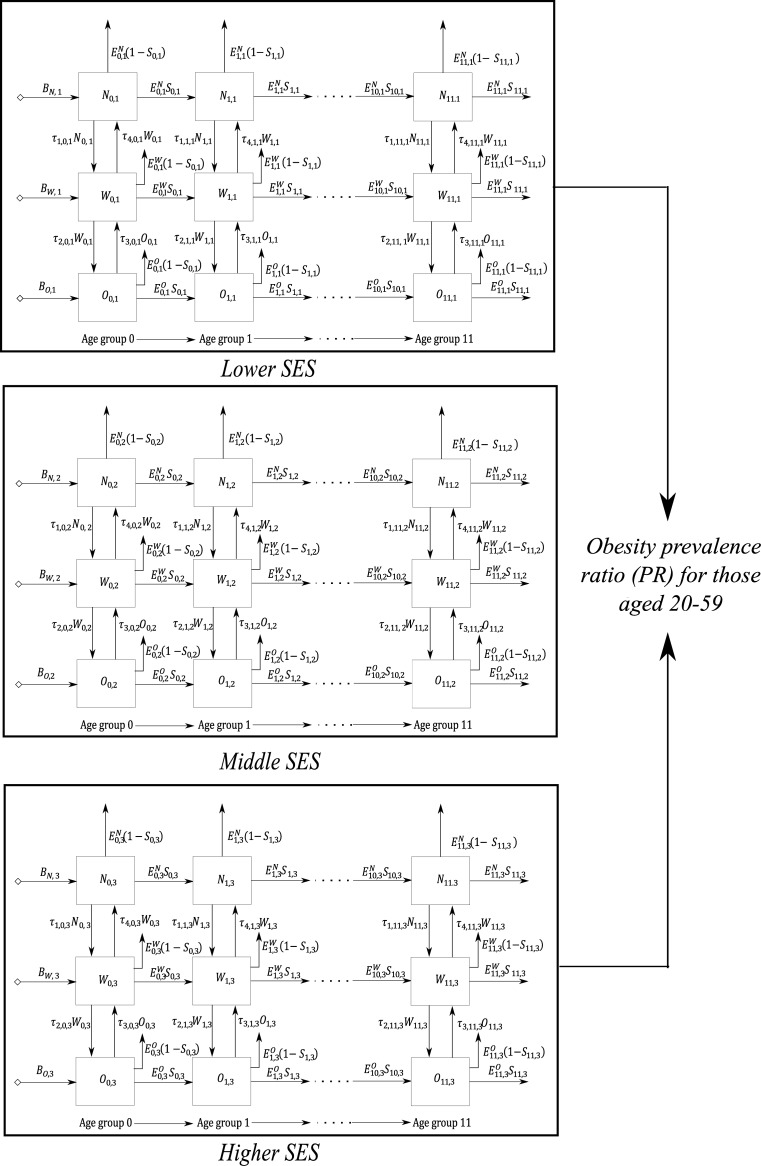
Overview of the SD model structure stratified by SES.

To apply the same SD model, but stratified by BMI and SES groups, we used the World Bank’s Wealth Index (WI) [45] as our SES indicator because it allows categorization of the population into socio-economic groups, captures quality of life markers, and is validated. This index takes into account assets such as a radio, television, refrigerator, car or motorcycle as well as household dwelling characteristics such as drinking water source, flooring quality, or toilet installation [46]. We grouped the WI categories into three socio-economic groups: the lowest and second quintiles, the middle quintile, and the fourth and highest quintiles. Fig 2 shows a global view of the SD model structure by SES group. A full description of the SD model by SES is available in S3 Appendix.

To assess if the population are experiencing a nutritional transition where the burden of obesity is changing towards the lower SES population, we compared the lowest SES group with the highest SES group through the obesity prevalence ratio (PR) for individuals aged 20 to 59 years using the following equation:

PR=∑i=411Oi,1∑i=411(Ni,1+Wi,1+Oi,1)∑i=411Oi,3∑i=411(Ni,3+Wi,3+Oi,1) (20)

where *N*_*i*,*1*_, *W*_*i*,*1*_, and *O*_*i*,*1*_ represent all *not overweight*, *overweight*, and *obese* individuals with a lower SES, respectively, in age group *i*, and *N*_*i*,*3*_, *W*_*i*,*3*_, and *O*_*i*,*3*_ represent all *not overweight*, *overweight*, and *obese* individuals of a higher SES, respectively, in age group *i*. A PR value greater than one is obtained if the obesity prevalence rate of the lower SES group is greater than the obesity prevalence rate of the higher SES group, indicating that the burden of obesity is greater among adults of the lower SES.

To calibrate the model for each BMI and SES group, we estimated the TRs between BMI categories by age group and SES using data from the ENDS of 2005 and 2010. We calculated the TRs using the same heuristic described in subsection 2.2 (Table 3). The same heuristic was then generalized by subdividing each age group into three BMI and SES groups (S3 Appendix). Given the purpose of the study and aims of the proposed SD model, we assumed that individuals did not transition between SES groups but instead remained in the same SES group throughout the life of the simulation.

## Results

### Model of nutritional stage dynamics by age

**Fig 3:**
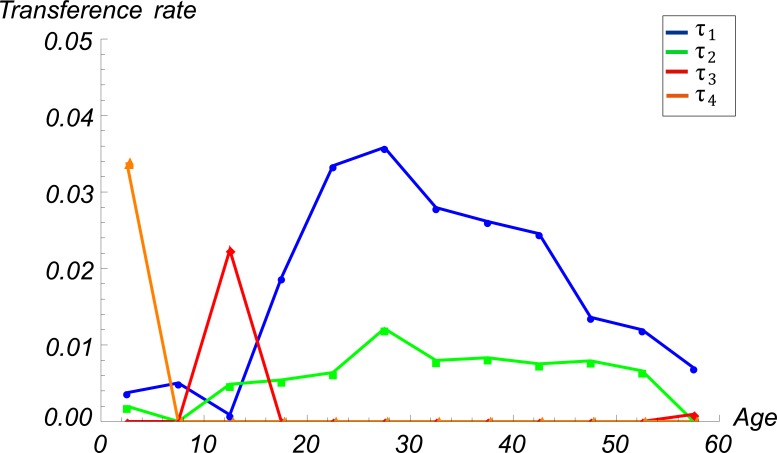
Estimated transference rates between BMI categories by age group. *Not overweight* to *overweight* (*τ*_*1*_), *overweight* to *obese* (*τ*_*2*_), *obese* to *overweight* (*τ*_*3*_), and *overweight* to *not overweight* (*τ*_*4*_).

Our estimates of the TRs between BMI categories by age group showed that the Colombian population is generally moving towards overweight and obesity. The only encouraging exceptions are children aged 0–4 and 10–14 years, who are transitioning from the *overweight* to *not overweight* (*τ*_*4*_) and from the *obese* to *overweight* (*τ*_*3*_) categories, respectively. The largest TRs from *not-overweight* to *overweight* (*τ*_*1*_) and from *overweight* to *obese* (*τ*_*2*_), ranging from 0.026 to 0.036 and from 0.006 to 0.012, respectively, occurred in individuals aged 20–39 years. The Individuals in the age group 40 to 59 year- are also shifting towards the *overweight* (*τ*_*1*_) and *obese* (*τ*_*2*_) categories, but the corresponding TRs were lower (Fig 3 and Table 2). The retention rates of individuals by BMI category and age group *i (α*_*1*,*i*_, *α*_*2*,*i*_, and *α*_*3*,*i*_), which correspond to the fractions of individuals who remain in the same BMI category between 2005 and 2010, were closed to 1.

**Fig 4:**
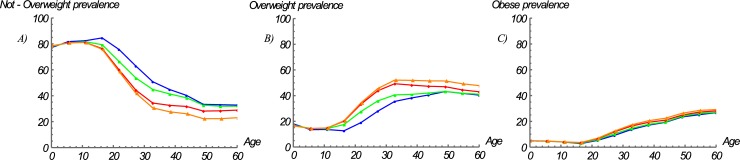
Estimated prevalence rates by BMI category over time. (A) *Not overweight*, (B) *overweight*, and (C) *obese*. Blue = 2005; Green = 2010; Red = 2020; Orange = 2030.

Using the estimated TRs, we modelled the nutritional stage dynamics from 2005 to 2030. In agreement with the TRs results, adults are moving towards the overweight and obese categories. The simulation results show that children aged 0 to 14 years will show no major changes in their BMI category distribution over time (Fig 4A). However, the prevalences of overweight and obesity increased from 12.6% and 2.8% in 2005 to 20.5% and 3.6% in 2030 for individuals aged 15 to 19 years (Fig 4B and 4C), respectively, and from 34.2% and 15.7% in 2005 to 47.7% and 19.2% in 2030 for adults aged 20 to 59 years (Fig 4C), respectively. Furthermore, the results show that the greatest increments between 2005 and 2030 occurred in individuals aged 20 to 39 years, with the prevalence of overweight increasing from 18.9 to 34.3 for individuals 20 to 24 years, from 27.9 to 45.6 for individuals aged 25 to 29 years, from 35.5 to 52.1 for individuals aged 30 to 34 years, and from 38.2 to 51.9 for individuals aged 35 to 39 years (Fig 4B). In contrast, the greatest increments in the prevalence of obesity, were observed in individuals aged 25 to 39 years, with increases from 9.2 to 12.5 for individuals aged 25 to 29 years, from 13.71 to 17.31 for individuals aged 30 to 34 years, and from 17.0 to 20.1 for individuals aged 35 to 39 years (Fig 4C).

### Model of nutritional stage dynamics by age and SES

**Fig 5:**
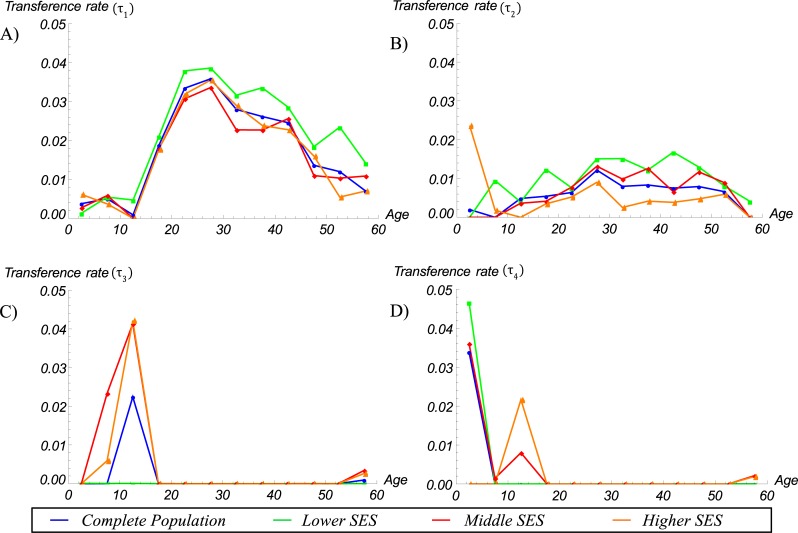
Estimated transference rates between BMI categories by age and SES. (A) *Not overweight* to *overweight* (*τ*_*1*_), (B) *overweight* to *obese* (*τ*_*2*_), (C) *obese* to *overweight* (*τ*_*3*_), and (D) *overweight* to *not overweight* (*τ*_*4*_).

We then estimated the TRs between BMI categories by age group and SES separately and found that the burden of overweight and obesity in Colombia is shifting towards those of lower SES (Fig 5A and 5B, Table 3). In particular, the TRs towards *overweight* (*τ*_*1*_) and *obese* (*τ*_*2*_) categories have increased more rapidly among individuals with a lower SES (mean *τ*_*1*_ = 0.0215 and mean *τ*_*2*_ = 0.0098) than among individuals with a middle (mean *τ*_*1*_ = 0.0162 and mean *τ*_*2*_ = 0.0065) or higher SES (mean *τ*_*1*_ = 0.0166 and mean *τ*_*2*_ = 0.0054) (Fig 5A and 5B, Table 3). However, in children aged zero to four years, these TRs (*τ*_*1*_ and *τ*_*2*_) have increased more rapidly among those with the highest SES than among those with a lower SES (Fig 5A and 5B, Table 3).

Unfortunately, the TRs from *obese* to *overweight* (*τ*_*3*_) and from *overweight* to *not overweight* (*τ*_*4*_) were almost zero for individuals aged 15 to 54 years in all three SES groups. For children aged five to 14, however, the TRs from *obese* to *overweight* and *overweight* (*τ*_*3*_) to *not overweight* (*τ*_*4*_) have increased more rapidly in the middle and higher SES groups than in the lower SES groups (Fig 5C and 5D, Table 3). Conversely, among children aged zero to four years, the TRs from *overweight* to *not overweight* (*τ*_*4*_) increased more rapidly in the lower SES group than in the other SES groups (Table 3).

**Fig 6:**
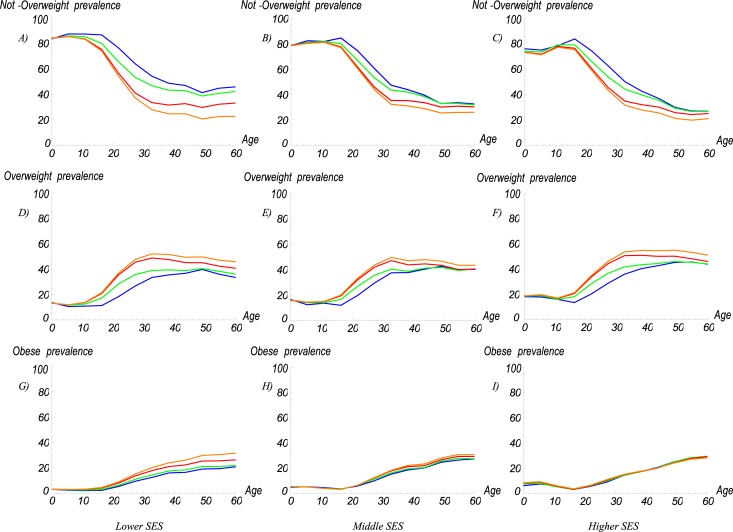
Estimated prevalence rates of BMI categories by age and SES groups. (A) *Not overweight*, lower SES; (B) *not overweight*, middle SES; (C) *not overweight*, higher SES; (D) *overweight*, lower SES; (E) *overweight*, middle SES; (F) *overweight*, higher SES; (G) *obese*, lower SES; (H) *obese*, middle SES; and (I) *obese*, higher SES. Blue = 2005; Green = 2010; Red = 2020; Orange = 2030.

The simulation results also showed that the BMI category distribution of the Colombian population differs among different age and SES groups over time (Fig 6). Aligned with both the results of the TRs by SES and the simulations for the entire urban population, the most notable changes were observed in adults. That is, the prevalence of overweight among adults with a lower, middle, and higher SES increased from 30.7% to 47%, 34.3% to 44.3%, and 36.4% to 50%, respectively, between 2005 and 2030. In obese adults, the differences between SES groups were even more striking, with increases from 13% to 21.3% for lower SES and from 16.3% to 19.9% for middle SES adults. Higher SES group on the other hand experienced a relatively small increase from 17.2% to 17.8% (Fig 6G, 6H and 6I).

**Fig 7:**
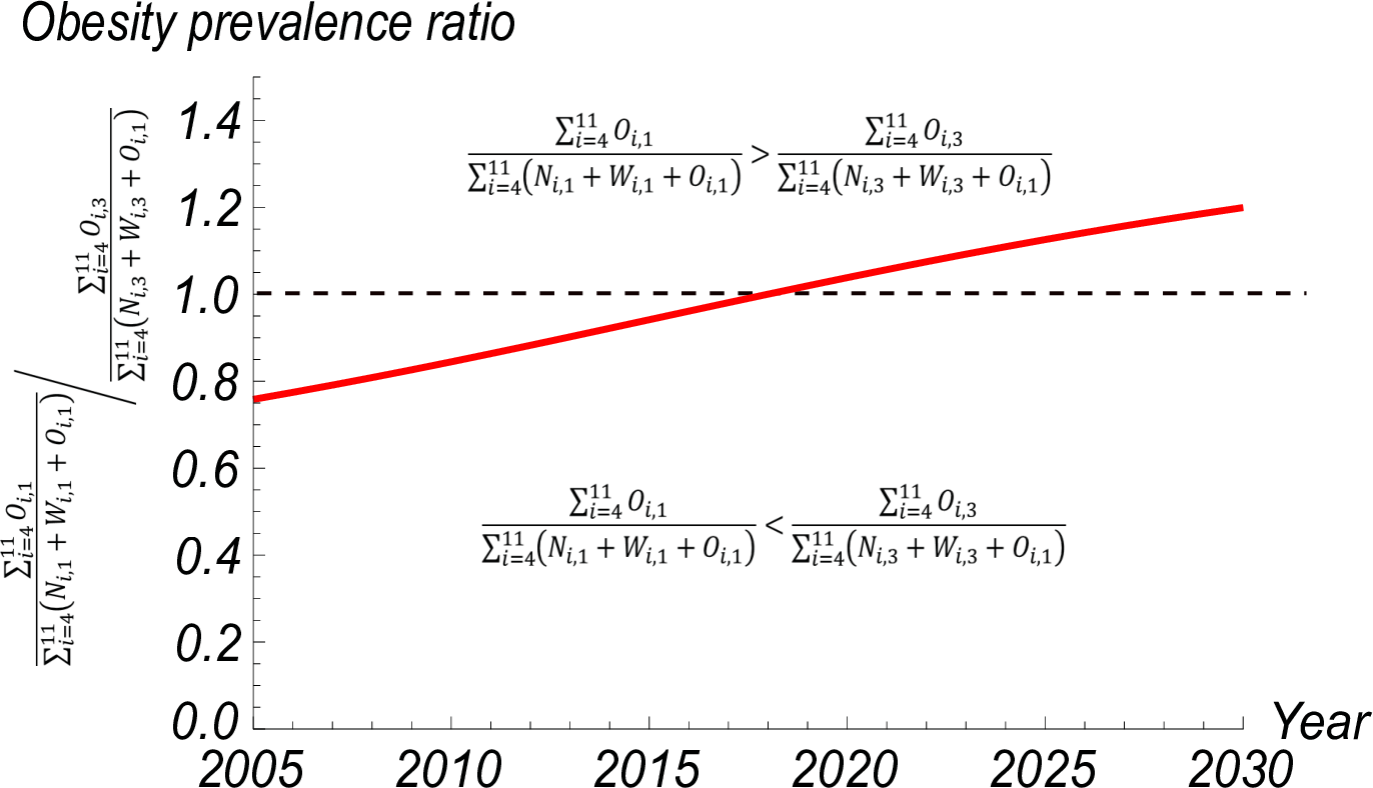
Obesity prevalence ratio amongst urban adults aged 20 to 59 years.

Based on the obesity PR predicted by this SD model, if the estimated TRs between BMI categories by age and SES remain unchanged for the life-course of the simulation (i.e., to the year 2030), then the burden of obesity among adults will continue to shift towards those with a lower SES. As shown in Fig 7 the PR of obesity in the lower SES group in 2018 will be approximately equal to that of the higher SES group.

### Utility of the simulation to assess the effect of future policy interventions

This SD model can be used to conduct simulations aiming to project the potential effects of policy interventions on the prevalence of overweight and obesity stratified by age and SES. For example, we simulated the potential impact of certain action lines from Law 1355 of 2009, which defines obesity and NCDs as a public health priority. Among its prevention and control measures, this law stipulates that “the public and private schools of the country should adopt a healthy food programme and promote physical activity (PA)”. To simulate the potential impact of this law, we assess two scenarios:

#### Scenario 1

**Fig 8:**
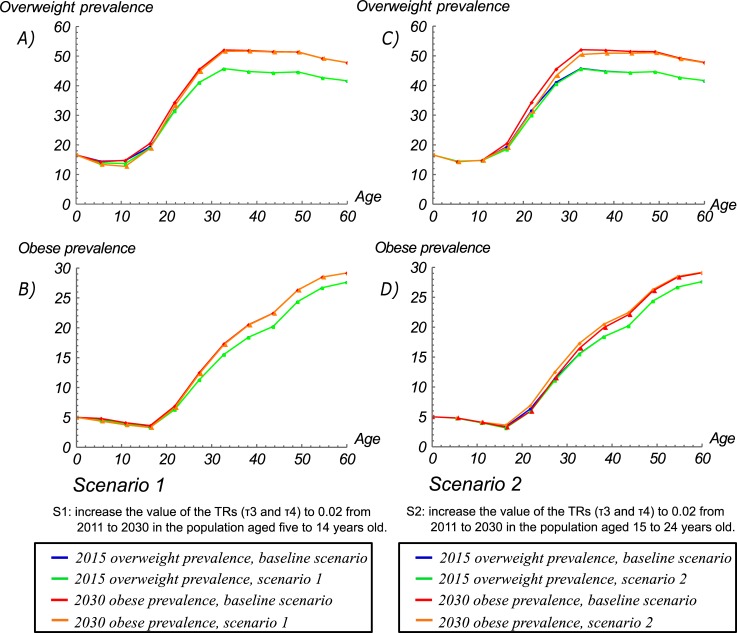
Estimated prevalence rates of BMI categories, by age group, under two scenarios. (A) Overweight prevalence rates under scenario 1; (B) obese prevalence rates under scenario 1; (C) overweight prevalence rates under scenario 2; (D) obese prevalence rates under scenario 2.

We assume that if these actions are effective and fully implemented through PA promotion and healthy nutritional initiatives, this could result in an increase in the TRs from *obese* to *overweight* (*τ*_*3*_) and from *overweight* to *not overweight* (*τ*_*4*_) in the population aged five to 14 years. We used a conservative estimate which assumed that the implementation of such initiatives could increase the TRs (*τ*_*3*_ and *τ*_*4*_) to 0.02, which is lower than the increment in meeting physical activity recommendations (0.03) between 2005 and 2010 reported in the National Nutrition Survey [47]. The results of the simulations obtained by increasing the value of these TRs (*τ*_*3*_ and *τ*_*4*_) to 0.02 from 2011 to 2030 show that compared with the baseline scenario, the prevalences of overweight and obesity could decrease by 4.3% and 2.5% by 2015 and by 9% and 9.5% by 2030, respectively, among individuals aged 5 to 19 years. For those aged 20 to 59 years, the corresponding decreases are 0% by 2015 and 0.8% and 0.5% by 2030, respectively (Fig 8A and 8B). In particular, the results showed that the greatest changes, could occur in individuals aged 10 to 24, with the prevalence of overweight decreasing from 14.8 to 12.7 for individuals aged 10 to 14 years, from 20.5 to 18.8 for individuals aged 15 to 19 years, and from 34.4 to 33.2 for individuals aged 20 to 24 years, by 2030 (Fig 8A).

#### Scenario 2

On the other hand, the results of the simulation of the same effect in the same TRs (*τ*_*3*_ and *τ*_*4*_) for individuals aged 15 to 24 years (the age period where there is an important increment in the TRs) showed on a greater impact on adults. The results obtained by increasing the value of these TRs (*τ*_*3*_ and *τ*_*4*_) to 0.02 from 2011 to 2030 show that compared with the baseline scenario, the prevalences of overweight and obesity could decrease by 0.9% and 0% by 2015 and by 3% and 4.8% by 2030, respectively, for individuals aged 5 to 19 years; for adults aged 20 to 59 years, the reduction is 0.7% and 0.6% by 2015 and 2.7% and 3.1% by 2030, respectively (Fig 8C and 8D). In particular, the results showed that the greatest changes, could occur in individuals aged 15 to 34 years, with the prevalence of overweight decreasing from 20.5 to 19.1 for individuals aged 15 to 19 years, from 34.4 to 31.3 for individuals aged 20 to 24 years, from 45.6 to 43.4 for individuals aged 25 to 29 years, and from 52.1 to 50.5 for individuals aged 30 to 34 years, by 2030 (Fig 8C).

## Discussion

Because the growing obesity epidemic has emerged as an even greater public health challenge [1,2], it is becoming crucial to increase our understanding of nutritional stage dynamics, particularly with respect to population age and SES. Aligned with this aim, our study applied a systemic approach which shows that urban Colombian adults are experiencing a nutritional transition in which the burdens of overweight and obesity are shifting towards those with a lower SES. This study constitutes the first use of TR indicators to show that the TRs from *not overweight* to *overweight* and from *overweight* to *obese* have increased more rapidly among individuals with a lower SES than among those in the other SES groups. Furthermore, the results showed that the prevalences of obesity are increasing faster in the lowest SES group compared to the highest SES group. Particularly notable are the TRs from *not overweight* to *overweight* and from *overweight* to *obese* for individuals aged 20 to 39 years in the lowest SES group, which are increasing more rapidly than the corresponding TRs for the other age and SES groups. These findings provide evidence that Colombian adults are experiencing a nutritional transition that is highly dependent on the SES, which suggest that policy interventions should be differentiated accordingly.

Our results are consistent with those documented by previous studies. Jones-Smith *et al*. [6] showed that 31% (using wealth as the SES indicator) or 54% (using education as the SES indicator) of countries observe faster overweight growth rates in the lower SES groups than the higher SES groups. Additionally, faster overweight growth rates in the lower wealth groups were found to be positively associated with the gross national product (GNP). Similarly, Monteiro *et al*. [5], who explored the relationship among obesity, SES, and level of economic development among adults in developing countries, established that women with a lower SES are more likely to be obese than women with a higher SES, and that this likelihood augments as GNP increases. In males specifically, as GNP increases, individuals from lower SES groups tend to lose their protection against obesity. Similarly, Dinsa *et al*. [18] found a negative relationship between SES and obesity in women and that this association becomes mixed for men in middle-income countries (MICs).

It is encouraging that children aged 0 to 14 years with a middle or high SES, are the only age groups that are transitioning from the *obese* to *overweight* and from the *overweight* to *not overweight* categories. We hypothesize that this result could be explained in part by several factors. First, children from middle to high SES may have more access to PA education at school and out of school activities and more access to healthy food. Second, the actions taken by the Colombian government to improve PA and prevent obesity and non-communicable diseases might have played a role. For example, at the national level, the government established Law 1355/2009 (known as the obesity law), which stipulates that schools should ensure fruit and vegetable availability, implement food education programmes, and regulate the consumption of highly caloric foods and beverages. Similarly, the government has enacted various regulations (Laws 115/1994 and 934/2004) that mandate physical education programmes in schools for all grades. Furthermore, Colombia has the *Ciclovías Recreativas*, which is a mass recreational program with national coverage, implemented over a decade ago to promote PA. At the departmental level, Law 715/2001 tasks government departments with promoting PA. Since the law’s enactment, most departments in Colombia prioritize PA in children and youths in their departmental development plans. Nevertheless, the effectiveness of these policies and programmes by SES must be evaluated [48], and simulations similar to those illustrated in this paper must be validated through policy effectiveness analyses.

The findings of our study must be considered with a few limitations in mind. First, given the lack of longitudinal data for tracing individual growth by BMI category and age, we estimated the TRs between BMI categories using only survey data for 2005 and 2010 obtained from the ENDS. Future studies should include more time points to validate the model. Second, due to the lack of studies in Colombia that estimate mortality rates by BMI category among adults, we used the same mortality rates for each BMI category. Future studies should incorporate the effect of mortality rate associated with each BMI category and explore how these impact model dynamics including the simulated prevalence rates by BMI category among adults. Third, we did not include individuals older than 60 years in the model given the absence of data that would enable the reliable estimation of parameters for older age groups. Furthermore, the results of the estimated TRs for individuals aged 50 to 59 years showed that the number of people who changed BMI categories was small, and that the TRs for these age groups are decreasing. This need for available data underscores the importance of the government continuing to collect complete data on BMI and health-related behaviours (e.g., PA), by age, SES, sex, and region for the entire population, in upcoming Demographic and Health Surveys. Another limitation relates to our assumption that the TRs are uniform across each five-year age groups and that individuals do not transition between SES groups. Our results show that the SD model is capable of reproducing the dynamics that give rise to the prevalence rates by BMI category, age, and SES observed in the ENDS data. Nevertheless, although we showed that the obesity dynamics vary among SES and age groups, the behavioural and environmental variables that explain the TRs differences between BMI categories by age and SES are not included in the model. Future studies should focus on understanding the effects of various factors, such as the sex, PA level, sedentary behaviours and consumption of processed food and sugary drinks on nutritional stage dynamics.

Despite the aforementioned limitations, our study has a number of important strengths. First, we propose a new heuristic to estimate the TRs between BMI categories by age and SES that could be expanded in the future to other socioeconomic attributes. Second, we assessed the nutritional transition by SES in LMICs using a complex systems approach. Specifically, we propose a new population-level SD model that may be used to understand obesity dynamics by age and SES. Third, we used a large collected data (ENDS), which included a sample covering a wide range of socioeconomic data and objective BMI measurements. Finally, we used the WI, which is an indicator of long-term economic circumstances, as our SES indicator for categorization of the population into SES groups.

In summary, we observed two patterns within the nutritional transition phenomenon in Colombia. First, the urban Colombian population, similar to that other MICs, is experiencing an SES-related nutritional transition in which the population aged 20 to 39 years has the highest TRs to unhealthy weight. Second, and as a reflection of SES disparities, the only age groups that are moving from the *obese* to *overweight* and from the *overweight* to the *not overweight* categories are children and adolescents aged 0 to 14 years from the middle and high SES groups.

## Conclusions

To the best of our knowledge this study constitutes the first assessment of the SES-related obesity dynamics in a LMIC using a complex systems approach. We proposed a heuristic to estimate the TRs and a population-level SD model to assess and model the nutritional stage dynamics of an urban population by age and SES, over time. The TR indicators were constructed, and this study constitutes the first use of these indicators to provide information regarding the fraction of individuals who move between BMI categories stratified by age and SES.

The model results, which have implications for the targeting of interventions in Colombia, indicate that the most vulnerable and poorest groups display higher TRs to overweight and obese, whereas wealthiest groups show the highest prevalences of overweight and obesity. Despite this, the main public health policies in Colombia targeting the lowest SES population are related to undernutrition and integrated policies for the whole spectrum of malnutrition are not effectively implemented. We also found that the prevalence of overweight and obesity among children aged 0 to 14 years do not follow the patterns observed in other middle income countries in Latin America.

The model proposed in this paper offers policymakers a useful tool to better monitor nutritional transitions among diverse segments of the population. In particular, the model could be employed to estimate the TRs between BMI categories by age and SES, and to help identify which subgroups of the population should be targeted. The model could also be used to monitor the impact of health interventions implemented to prevent the development of overweight and obesity related to age and SES. Finally, with proper adaptations this model could be used for assessing SES-related nutritional transitions in other LMICs.

## Supporting information

S1 Appendix Calibration and validation of the model. This file shows the different methods used to assess the suitability of the proposed system dynamics model.
(DOCX)

S2 Appendix Parameters and data used in the model. This file describes the data and parameters used in the system dynamics model and the heuristic.
(DOCX)

S3 Appendix System dynamics model by SES. This file describes the system dynamics model stratified by socioeconomic status groups.
(DOCX)
