# Supplementary material for: Towards a novel model for studying the nutritional stage dynamics of the Colombian population by age and socioeconomic status
Source: PLoS One. 2018 Feb 8;13(2):e0191929. doi: 10.1371/journal.pone.0191929 (PMC5805245; doi:10.1371/journal.pone.0191929)
Supplement: S1 Appendix — This file shows the different methods used to assess the suitability of the proposed system dynamics model. (DOCX) [file pone.0191929.s001.docx]

**Supplementary Information 1**

**Supplement to: Towards a novel model for studying the nutritional stage dynamics of the Colombian population by age and socioeconomic status**

**Table of contents**

**Section 1. Calibration and validation of the model** 3

**Integration error method** 3

**Parameter assessment method** 3

**Extreme conditions method** 5

**Sensitivity analysis method** 14

**Behaviour reproduction method** 14

**References** 17

**Section 1. Calibration and validation of the model**

We developed five tests to assess the suitability of the proposed system dynamics (SD) model to investigate nutritional stage dynamics within the Colombian urban population by both age and socio-economic status (SES) [1]: integration error, which tests whether the results of the model are sensitive to different numerical integration methods and time steps; parameter assessment, which tests whether the parameter values are consistent with relevant descriptive and numerical data; extreme conditions, which tests whether the behaviour of the model is consistent for extreme values; sensitivity analysis, which tests the numerical behaviour and policy sensitivity of the model; and behaviour reproduction, which tests whether the model reproduces the behaviour of interest in the system. In general, we assess the suitability of the SD model only for the entire population because we used the same SD model to assess the nutritional stage dynamics of the population by SES (according to the purpose of the data and definition of the proposed SD model, we assume that individuals do not transition between SES groups).

## **Integration error method**

The SD models are structured in continuous time and simulated by numerical integration. The modeller must identify a numerical integration method and time step that generate results accurate enough for the purpose of the model [1]. We used the integration error method to test whether the SD model is sensitive to variations in the time step or integration method. Ideally, the results of the model should not be sensitive to the integration method nor the choice of time step. We tested different methods of integration (Runge kutta 4, Runge kutta 2, and Euler’s methods) and time steps (0.01, 0.25 and 1) using iThink 9.0.2. The results of this test showed that the SD model is insensitive to both the choice in time step and integration method. That is, the behaviour pattern of the model does not change when we use different time steps or integration methods (Fig 1). For the rest of the simulations, we used a time step of 0.25 and the Euler’s integration method.

## **Parameter assessment method**

The parameter assessment method to estimate and calibrate the transference rates (TRs), which correspond to the fraction of individuals within each age group that move between body mass index (BMI) categories, is described in the main text of the article. The results of the parameter assessment showed that the quadratic differences between the prevalence rates by BMI category reported by ENDS in 2010, and the estimated 2010 prevalence rates by the proposed heuristic, were less than 5% for each age and SES group (Table 1). These values indicates that the prevalence rates predicted by the heuristic closely replicate the prevalence rates observed in 2010 (historical data).

**Fig 1. Estimated prevalences by BMI category over time produced by different integration methods and time steps.** (A, B, C) Runge kutta 4 method and time step of 0.01; (D, E, F) Runge kutta 2 method and time step of 0.01; (G, H, I) Euler’s method and time step of 0.01; and (J, K, L) Euler’s method and time step of 0.25.

**Table 1. Quadratic differences between the prevalence rates by BMI categories reported by the ENDS in 2010 and the estimated 2010 prevalence rates using the heuristic**

| **Age group** | ***Entire population***  ***(%)*** | ***Lower SES***  ***(%)*** | ***Middle SES***  ***(%)*** | ***Higher SES***  ***(%)*** |
| --- | --- | --- | --- | --- |
| 0-4 | 2.29 | 1.97 | 2.71 | 2.66 |
| 5-9 | 1.10 | 0.54 | 2.05 | 3.23 |
| 10-14 | 0.67 | 0.44 | 1.25 | 1.52 |
| 15-19 | 3.74 | 4.78 | 5.23 | 3.21 |
| 20-24 | 3.12 | 4.25 | 3.00 | 3.08 |
| 25-29 | 3.30 | 3.99 | 4.20 | 3.12 |
| 30-34 | 1.82 | 4.52 | 2.42 | 2.11 |
| 35-39 | 1.34 | 2.60 | 2.20 | 1.01 |
| 40-44 | 1.15 | 2.99 | 2.02 | 1.44 |
| 45-49 | 0.79 | 2.48 | 2.90 | 1.12 |
| 50-54 | 0.58 | 2.91 | 1.87 | 1.09 |
| 55-59 | 1.22 | 3.29 | 1.95 | 1.72 |

## **Extreme conditions method**

We used the extreme conditions method to test whether the SD model is robust under extreme conditions. A model is robust to extreme conditions if the model is capable of generating realistic behaviour, no matter how extreme the inputs (parameters) or policies imposed [1]. We assessed extreme values (0 and 1) for the TRs, mortality rates, and fertility rates and analysed their effect on the estimated prevalence rates by BMI category.

**Extreme values for the TRs**

For each age group, we developed two tests. First, we specified that the TR from not overweight to overweight *τ_1_* and from overweight to obesity *τ_2_* take the values of 0 or 1. Second, we defined the TRs from obesity to overweight *τ_3_* and from overweight to not overweight *τ_4_*, such that they take the values of 0 or 1. We used the sensitivity specs tool in iThink 9.0.2 to develop these tests. Here, we only report the assessment of extreme values for TRs of the age groups 15-19, 20-24, and 25-29 (these age groups have the greatest increments in the TR towards overweight and obesity) to show the robustness of the SD model (Figs. 2, 3, 4, 5). The results of the tests for the other age groups were similar to the results reported in Figs. 2, 3, 4, 5.

The results showed that the SD model is robust and that the behaviour pattern of the model was consistent with expected results for the estimated prevalence rates by BMI category. For example, when *τ_1_*= 1 and *τ_2_* = 1 for the age group 15-19, the simulation results, aggregated across ages to report prevalence rates for age groups (5–19 and 20–59), showed that for age group 5-19 the prevalence of overweight increases in the first years but then stabilizes. However, how the *τ_2_* is also taken as 1, the highest increase occurs in the prevalence of obesity. The prevalence of the not-overweight category decreases over time due to the increase of *τ_1_* and *τ_2_* (Fig. 2). For adults aged 20-59, the prevalence of overweight increases in the first years but then it decreases over time, while the overall prevalence of obesity increases over time. In contrast, when *τ_1_*= 0 and *τ_2_* = 0 for the age group 15-19, the simulation results showed that the prevalence of overweight and obesity for children and adolescents aged 5-19 have a lower increase than the normal scenario since *τ_1_* and *τ_2_* for the age groups 5-9 and 10-14 do not change for this test. Furthermore, the effects that could be generated on the estimated prevalence rates by BMI category for adults aged 20-59 were not significant (Fig. 2).

**Fig 2. Estimated prevaleneces by BMI category over time when changing *τ_1_* and *τ_2_* of age group 15-19 years between 0 and 1 in intervals of 0.1.** (A, B, C) Population aged 5-19 years; (D, E, F) Population aged 20-59 years. Each line showed the results of the simulation for the prevalences of not-overweight, overweight, and obese, respectively, when *τ_1_* and *τ_2_* of the age group 15-19 years take values between 0 and 1 in intervals of 0.1. For example, line 1 shows the results when *τ_1_* and *τ_2_* take, simultaneously, the value of zero; line 6 shows the results when *τ_1_* and *τ_2_* take, simultaneously, the value of 0.5; line 11 shows the results when *τ_1_* and *τ_2_* take, simultaneously, the value of 1.

**Fig 3. Estimated prevaleneces by BMI category over time when changing *τ_3_* and *τ_4_* of age group 15-19 years between 0 and 1 in intervals of 0.1.** (A, B, C) Population aged 5-19 years; (D, E, F) Population aged 20-59 years. Each line showed the results of the simulation for the prevalences of not-overweight, overweight, and obese, respectively, when *τ_3_* and *τ_4_* of the age group 15-19 years take values between 0 and 1 in intervals of 0.1. For example, line 1 shows the results when *τ_3_* and *τ_4_* take, simultaneously, the value of zero; line 6 shows the results when *τ_3_* and *τ_4_* take, simultaneously, the value of 0.5; and line 11 shows the results when *τ_3_* and *τ_4_* take, simultaneously, the value of 1.

**Fig 4. Estimated prevaleneces by BMI category of the adults aged 20-59 years over time when changing *τ_1_* and *τ_2_* between 0 and 1 in intervals of 0.1.** (A, B, C) when changing *τ_1_* and *τ_2_* of the age group 20-24 years; (D, E, F) when changing *τ_1_* and *τ_2_* of the age group 25-29 years. Each line showed the results of the simulation for the prevalences of not-overweight, overweight, and obese of the adults aged 20-59 years, respectively, when *τ_1_* and *τ_2_* of the age group (20-24 or 25-29 years old age group) take values between 0 and 1 in intervals of 0.1. For example, line 1 shows the results when *τ_1_* and *τ_2_* take, simultaneously, the value of zero; line 6 shows the results when *τ_1_* and *τ_2_* take, simultaneously, the value of 0.5; line 11 shows the results when *τ_1_* and *τ_2_* take, simultaneously, the value of 1.

**Fig 5. Estimated prevaleneces by BMI category of the adults aged 20-59 years over time changing *τ_3_* and *τ_4_* between 0 and 1 in intervals of 0.1.** (A, B, C) changing *τ_3_* and *τ_4_* of the age group 20-24 years; (D, E, F) changing *τ_3_* and *τ_4_* of the age group 25-29 years. Each line showed the results of the simulation for the prevalences of not-overweight, overweight, and obese of the adults aged 20-59 years, respectively, when *τ_3_* and *τ_4_* of the age group (20-24 or 25-29 years old age group) take values between 0 and 1 in intervals of 0.1. For example, line 1 shows the results when *τ_3_* and *τ_4_* take, simultaneously, the value of zero; line 6 shows the results when *τ_3_* and *τ_4_* take, simultaneously, the value of 0.5; and line 11 shows the results when *τ_3_* and *τ_4_* take, simultaneously, the value of 1.

**Extreme values for the mortality rates**

For each age group, we specified that the mortality rate take the values of 0 or 1. Here, we only report the assessment of extreme values for the mortality rates of the age groups 15-19, 20-24, and 25-29 to show the robustness of the SD model (Figs. 6, 7). The results of the tests for the other age groups showed similar patterns to the results reported in Figs. 6, 7.

The results showed that the SD model is robust and that the behaviour pattern of the model was consistent with expected results for the estimated prevalence rates by BMI category (Figs 6, 7). For example, when the mortality rate take the value of 1 for the age group 15-19, the simulation results, aggregated across ages to report prevalence rates for age groups (5–19 and 20–59), showed that compared with the baseline scenario (Fig 6A, 6B, 6C), the population size decreases over time due to amount of people that die in the age group 15-19 (Fig 6D). Additionally, the behaviour pattern of the model does not change in the age group 5-19 (Fig 6E). For the age group 20-59, the prevalence of obesity increases more rapidly over time (Fig 6F), compared with the baseline scenario, due to that there are a break of the ageing chain structure in the SD model. There are a relevant decreases in the individuals aged 0 to 19, who have the greatest prevalences of not overweight, that could mature to the age group 20 to 24. How the number of individuals who mature to the age group 20 to 59 years decreases and the individuals aged 20 to 59 years have the greatest prevalences of obesity in the 2005, the initial year of the simulation, the prevalences of obesity for the adults population increases over time. In contrast, when the mortality rate take the value of 0 for the age group 15-19, the simulation results showed that there are not relevant changes in the behaviour pattern of the model for the different age groups (this behaviour is consistent because the mortality rates used in the SD model are closed to zero) (Fig 7D, 7E, 7F). The results of the tests for the other age groups were similar to the results described before.

**Extreme values for the fertility rate**

We specified that the fertility rate take the values of 0 or 10 from 2005 to 2050, as extreme values. The results showed that the SD model is robust and that the behaviour pattern of the model was consistent with expected results for the estimated prevalence rates by BMI category (Fig 8). When the fertility rate take the value of 0, the simulation results, aggregated across ages to report prevalence rates for age groups (0-4, 5–19 and 20–59), showed that compared with the baseline scenario (Fig 8A, 8B, 8C, 8D), the population size decreases over time due to the births for each BMI category are zero (Fig 8E). Additionally, the behaviour pattern of the model does not change in the age groups 0-4 and 5-19 (Figs 8F, 8G). For the age group 20-59, the prevalence of obesity increases more rapidly over time, compared with the baseline scenario, due to that there are a break of the ageing chain structure in the SD model. How the births for each BMI category are zero, the number of individuals for each age group (stock) decreases over time. This behaviour affect, in the first way, the first age groups, who have the greatest prevalences of not overweight, so the number of individuals who mature to the next age groups decreases. How the number of individuals who mature to the age group 20 to 59 years decreases and the individuals aged 20 to 59 years have the greatest prevalences of obesity in the 2005, the initial year of the simulation, the prevalences of obesity for the adults population increases over time.

In contrast, when the fertility rate take the value of 10, the simulation results showed that the population size increases exponentially over time due to the births increases exponentially (from 2,6 to 19 million per year approximately) (Fig 8I). How the number of individuals in each age group (stocks), increases exponentially due to the increases in the births, and the TRs are less than 6%, the number of individuals per year that move between BMI categories are no significant compare with the size of each age group. Therefore, this generate that the prevalences of not overweight, overweight and obesity for the different age groups are almost not change over time (Figs 8J, 8K, 8L).

**Fig 6. Estimated prevaleneces by BMI category over time when the mortality rate take the value of 1.** For the baseline scenario: A) Population aged 0 to 59 years, B) Estimated prevalences by BMI category for the individuals aged 5 to 19 years, C) Estimated prevalences by BMI category for the individuals aged 20 to 59 years; when mortality rate of the age group 15-19 take the value of 1: D) Population aged 0 to 59 years, E) Estimated prevalences by BMI category for the individuals aged 5 to 19 years, F) Estimated prevalences by BMI category for the individuals aged 20 to 59 years; when mortality rate of the age group 20-24 take the value of 1: G) Population aged 0 to 59 years, H) Estimated prevalences by BMI category for the individuals aged 5 to 19 years, I) Estimated prevalences by BMI category for the individuals aged 20 to 59 years; when mortality rate of the age group 25-29 take the value of 1: J) Population aged 0 to 59 years, K) Estimated prevalences by BMI category for the individuals aged 5 to 19 years, L) Estimated prevalences by BMI category for the individuals aged 20 to 59 years,

**Fig 7. Estimated prevaleneces by BMI category over time when the mortality rate take the value of 0.** For the baseline scenario: A) Population aged 0 to 59 years, B) Estimated prevalences by BMI category for the individuals aged 5 to 19 years, C) Estimated prevalences by BMI category for the individuals aged 20 to 59 years; when mortality rate of the age group 15-19 take the value of 0: D) Population aged 0 to 59 years, E) Estimated prevalences by BMI category for the individuals aged 5 to 19 years, F) Estimated prevalences by BMI category for the individuals aged 20 to 59 years; when mortality rate of the age group 20-24 take the value of 0: G) Population aged 0 to 59 years, H) Estimated prevalences by BMI category for the individuals aged 5 to 19 years, I) Estimated prevalences by BMI category for the individuals aged 20 to 59 years; when mortality rate of the age group 25-29 take the value of 0: J) Population aged 0 to 59 years, K) Estimated prevalences by BMI category for the individuals aged 5 to 19 years, L) Estimated prevalences by BMI category for the individuals aged 20 to 59 years.

**Fig 8. Estimated prevaleneces by BMI category over time when the fertility rate take the values 0 or 10.** For the baseline scenario: A) Population aged 0 to 59 years, B) Estimated prevalences by BMI category for the individuals aged 0 to 4 years, C) Estimated prevalences by BMI category for the individuals aged 5 to 19 years, D) Estimated prevalences by BMI category for the individuals aged 20 to 59 years; when fertility rate take the value of 0 from 2005 to 2050: E) Population aged 0 to 59 years, F) Estimated prevalences by BMI category for the individuals aged 0 to 4 years, G) Estimated prevalences by BMI category for the individuals aged 5 to 19 years, H) Estimated prevalences by BMI category for the individuals aged 20 to 59 years; when fertility rate take the value of 10 from 2005 to 2050: I) Population aged 0 to 59 years, J) Estimated prevalences by BMI category for the individuals aged 0 to 4 years, K) Estimated prevalences by BMI category for the individuals aged 5 to 19 years, L) Estimated prevalences by BMI category for the individuals aged 20 to 59 years.

## **Sensitivity analysis method**

Sensitivity analysis methods test whether the results and conclusions of a simulation change according to the model purpose, when different parameters of the model are changed over a plausible range. In general, there are three types of sensitivity analysis methods: 1) numerical sensitivity, to test whether changes in parameters or assumptions of the model change the numerical values of the results; 2) behaviour mode sensitivity, to test whether changes in parameters or assumptions of the model change the behaviour patterns of the model; and 3) policy sensitivity, to test whether changes in parameters or assumptions of the model change the impact or desirability of a proposed policy [1]. We used a behaviour mode sensitivity analysis to test whether the behaviour pattern of the SD model changes when there are changes in the TRs. For each age group, we developed two sensitivity analyses. First, we specified that the TR from not-overweight to overweight *τ_1_* and from overweight to obesity *τ_2_* take values between 0 and 1. Second, we defined that the TRs from obesity to overweight *τ_3_* and from overweight to not-overweight *τ_4_* also take values between 0 and 1. Using a variation interval of 0.1, we employed the sensitivity specs tool in iThink 9.0.2 to develop this test. As in the previous calibration method, we only report the sensitivity analysis of TRs for the age groups 15-19, 20-24, and 25-29 to show the robustness of the results generated by the SD model (Figs. 2, 3, 4, 5). The results of the test for the other age groups are similar to the results reported in Figs. 2, 3, 4, 5.

The results of the behaviour mode sensitivity analysis for each age group showed that the behaviour pattern of the model is consistent with expected trends for the estimated prevalence rates by BMI category. For instance, the results of the sensitivity analysis for the age group 15-19, when *τ_1_* and *τ_2_* take values between 0 and 1, showed that the prevalence of overweight increases initially, however, when *τ_1_* and *τ_2_* are greater than 0.2, the prevalence of overweight increases in the first few years but then it stabilizes. Furthermore, the prevalence of obesity increases over time when *τ_1_* and *τ_2_* increase. Conversely, the prevalence of not-overweight decreases over time, due to increases in *τ_1_* and *τ_2_* (Fig. 2). A similar behaviour pattern occurs for adults aged 20-59. In this case, the effects produced by the increase in *τ_1_* and *τ_2_* for the age group 15-19 on the estimated prevalence rates by BMI categories were significant. Additionally, it is important to highlight that the aging chain structure of the SD model is able to capture effects that could generate changes in the TRs of the younger age groups on the estimated prevalence rates by BMI categories of older age groups over time (Figs. 2, 3, 4, 5).

## **Behaviour reproduction method**

The behaviour reproduction method test whether the SD model is capable of reproducing the behaviour of the system. There are different measures to assess model fit to data such as, the coefficient of determination R^2^, the mean absolute error, the mean square error, and a number of other statistical test [1]. We used a two sample Kolmogorov–Smirnov test [2] to validate whether the model has been able to reproduce the behaviour of the prevalence rates by age of each BMI category and SES group in the population. The two sample Kolmogorov–Smirnov test checks whether the two data samples come from populations with the same distribution. The null hypothesis is that the two data samples come from a populations with the same distribution. The National Demographic and Health Survey (ENDS) is only conducted every 5 years. For the first time in Colombia, in 2005 the National survey included weight and height for the entire population. Since then, only one survey has been conducted in 2010. Due to a lack of longitudinal data with which to trace the individual growth by BMI category and age, we can only validate the behaviour reproduction of the model for the year 2010.

We used a two sample Kolmogorov–Smirnov test function in Mathematica to compare the prevalence rates by age reported by ENDS in 2010 and the estimated 2010 prevalence rates by age using the SD model. The results of the tests show that the SD model is able to reproduce the 2010 prevalence rates by age for each BMI category and SES in the population (all p-values were greater than 0.05, the null hypothesis (Ho) cannot be rejected, therefore suggesting that the two data samples, for each level and BMI category, come from populations with the same distribution) (Table 2 and Fig. 9).

Table 2. Results of the Kolmogorov–Smirnov tests

| **Level** | **Item** | **p-value** |
| --- | --- | --- |
| Complete population | Non-overweight prevalence rates vs. estimated prevalence rates of non-overweight by age | 0.998 |
|  | Overweight prevalence rates vs. estimated prevalence rates of overweight by age | 0.998 |
|  | Obese prevalence rates vs. estimated prevalence rates of obesity by age | 0.869 |
| Lower SES | Non-overweight prevalence rates vs. estimated prevalence rates of non-overweight by age | 0.536 |
|  | Overweight prevalence rates vs. estimated prevalence rates of overweight by age | 0.998 |
|  | Obese prevalence rates vs. estimated prevalence rates of obesity by age | 0.536 |
| Middle SES | Non-overweight prevalence rates vs. estimated prevalence rates of non-overweight by age | 0.869 |
|  | Overweight prevalence rates vs. estimated prevalence rates of overweight by age | 1 |
|  | Obese prevalence rates vs. estimated prevalence rates of obesity by age | 0.998 |
| Higher SES | Non-overweight prevalence rates vs. estimated prevalence rates of non-overweight by age | 0.998 |
|  | Overweight prevalence rates vs. estimated prevalence rates of overweight by age | 0.998 |
|  | Obese prevalence rates vs. estimated prevalence rates of obesity by age | 0.998 |

**
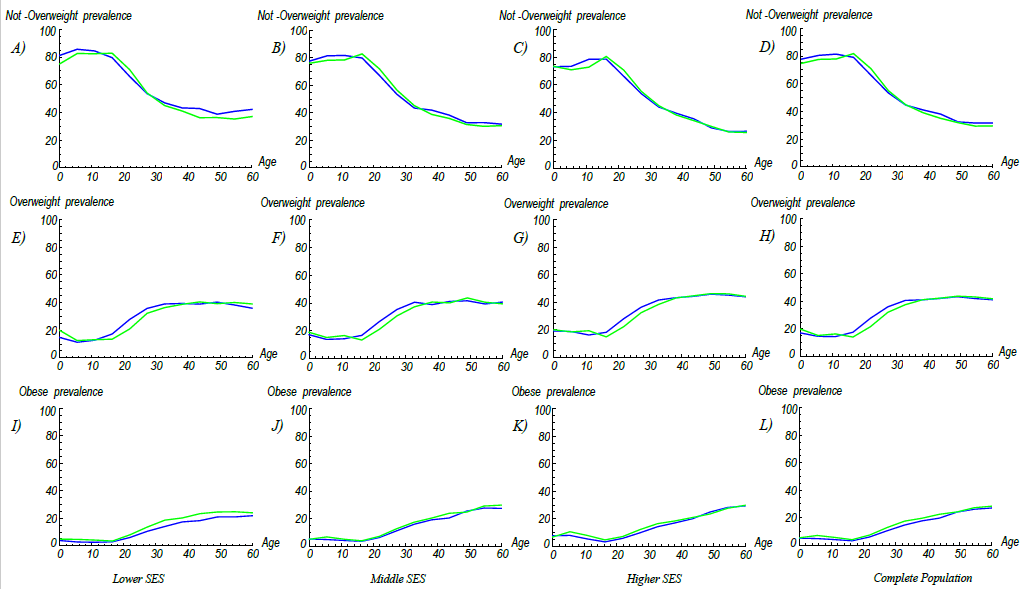
**

**Fig 9. Prevalence rates by age reported by ENDS in 2010 vs. estimated 2010 prevalence rates by age using the SD model by BMI categories and SES.** (A, E, I) Lower SES; (B, F, J) Middle SES; (C, G, K) Higher SES; and (D, H, L) Entire population. Blue= Prevalence rates by age reported by ENDS in 2010; Green= Estimated prevalence rates by age using the SD model.

**References**

1. Sterman JD. Business Dynamics: Systems Thinking and Modeling for a Complex World. USA: McGraw-Hill/Irwin; 2000.

2. Corder GW, Foreman DI. Nonparametric Statistics: A Step-by-Step Approach. 2 edition. Hoboken, New Jersey: Wiley; 2014.
