# Supplementary material for: Towards a novel model for studying the nutritional stage dynamics of the Colombian population by age and socioeconomic status
Source: PLoS One. 2018 Feb 8;13(2):e0191929. doi: 10.1371/journal.pone.0191929 (PMC5805245; doi:10.1371/journal.pone.0191929)
Supplement: S2 Appendix — This file describes the data and parameters used in the system dynamics model and the heuristic. (DOCX) [file pone.0191929.s002.docx]

**Supplementary Information 2**

**Supplement to: Towards a novel model for studying the nutritional stage dynamics of the Colombian population by age and socioeconomic**

**Table of contents**

**Section 1. Data used to estimate the transference rates** 3

**Section 2. Data and model parameters used in the system dynamics models** 10

**References** 13

**Section 1. Data used to estimate the transference rates**

The following tables describe the data used to estimate the transference rates (TRs) by age, body mass index (BMI) category, and socioeconomic status (SES).

**Table 1. Prevalence rates by BMI category, age, and socioeconomic status (SES) in 2005 ^†^**

| **Age** | **Nutritional Status** | **Prevalence rates** | | | |
| --- | --- | --- | --- | --- | --- |
|  |  | **Lower SES** | **Middle SES** | **Higher SES** | **Complete Urban Population** |
| 0 | Not-overweight | 81.87 | 79.32 | 75.84 | 79.37 |
|  | Overweight | 13.93 | 15.11 | 19.10 | 15.80 |
|  | Obese | 4.20 | 5.57 | 5.06 | 4.84 |
| 1 | Not-overweight | 73.76 | 70.94 | 68.28 | 71.36 |
|  | Overweight | 21.70 | 22.56 | 24.24 | 22.68 |
|  | Obese | 4.53 | 6.50 | 7.47 | 5.96 |
| 2 | Not-overweight | 74.85 | 71.85 | 72.92 | 73.42 |
|  | Overweight | 21.52 | 21.28 | 19.57 | 20.84 |
|  | Obese | 3.64 | 6.86 | 7.51 | 5.74 |
| 3 | Not-overweight | 81.01 | 75.75 | 76.18 | 78.02 |
|  | Overweight | 15.65 | 19.74 | 17.09 | 17.23 |
|  | Obese | 3.33 | 4.51 | 6.73 | 4.75 |
| 4 | Not-overweight | 85.94 | 83.58 | 79.48 | 83.33 |
|  | Overweight | 11.59 | 14.32 | 15.90 | 13.66 |
|  | Obese | 2.46 | 2.11 | 4.63 | 3.01 |
| 5 | Not-overweight | 85.85 | 86.62 | 77.52 | 83.61 |
|  | Overweight | 12.76 | 8.80 | 17.45 | 13.03 |
|  | Obese | 1.39 | 4.58 | 5.03 | 3.36 |
| 6 | Not-overweight | 84.30 | 83.27 | 75.08 | 81.27 |
|  | Overweight | 14.09 | 12.73 | 17.61 | 14.77 |
|  | Obese | 1.62 | 4.00 | 7.31 | 3.96 |
| 7 | Not-overweight | 86.62 | 80.30 | 74.47 | 80.97 |
|  | Overweight | 10.22 | 16.36 | 16.41 | 13.88 |
|  | Obese | 3.16 | 3.35 | 9.12 | 5.15 |
| 8 | Not-overweight | 89.10 | 82.51 | 73.21 | 81.85 |
|  | Overweight | 8.06 | 10.89 | 20.42 | 13.07 |
|  | Obese | 2.84 | 6.60 | 6.37 | 5.08 |
| 9 | Not-overweight | 88.15 | 78.87 | 74.23 | 81.12 |
|  | Overweight | 9.63 | 14.34 | 16.56 | 13.15 |
|  | Obese | 2.22 | 6.79 | 9.20 | 5.72 |
| 10 | Not-overweight | 86.32 | 80.25 | 71.97 | 80.11 |
|  | Overweight | 10.54 | 12.65 | 19.08 | 13.80 |
|  | Obese | 3.14 | 7.10 | 8.96 | 6.09 |
| 11 | Not-overweight | 85.26 | 78.33 | 76.66 | 80.33 |
|  | Overweight | 13.16 | 16.00 | 17.29 | 15.38 |
|  | Obese | 1.58 | 5.67 | 6.05 | 4.28 |
| 12 | Not-overweight | 86.54 | 85.24 | 76.65 | 83.13 |
|  | Overweight | 11.97 | 11.81 | 16.47 | 13.33 |
|  | Obese | 1.50 | 2.95 | 6.89 | 3.54 |
| 13 | Not-overweight | 88.03 | 82.47 | 82.97 | 84.72 |
|  | Overweight | 9.57 | 13.31 | 12.93 | 11.79 |
|  | Obese | 2.39 | 4.22 | 4.10 | 3.50 |
| 14 | Not-overweight | 86.80 | 82.70 | 82.01 | 84.09 |
|  | Overweight | 11.74 | 14.88 | 14.75 | 13.60 |
|  | Obese | 1.47 | 2.42 | 3.24 | 2.31 |
| 15 | Not-overweight | 84.86 | 79.34 | 81.44 | 82.27 |
|  | Overweight | 13.32 | 16.53 | 16.17 | 15.12 |
|  | Obese | 1.83 | 4.13 | 2.40 | 2.61 |
| 16 | Not-overweight | 86.08 | 86.23 | 82.99 | 85.05 |
|  | Overweight | 12.50 | 9.42 | 14.93 | 12.46 |
|  | Obese | 1.42 | 4.35 | 2.09 | 2.49 |
| 17 | Not-overweight | 86.47 | 82.24 | 83.81 | 84.40 |
|  | Overweight | 11.22 | 14.49 | 12.95 | 12.70 |
|  | Obese | 2.31 | 3.27 | 3.24 | 2.89 |
| 18 | Not-overweight | 88.32 | 89.03 | 84.60 | 86.94 |
|  | Overweight | 10.32 | 8.62 | 12.37 | 10.78 |
|  | Obese | 1.35 | 2.35 | 3.02 | 2.28 |
| 19 | Not-overweight | 85.03 | 83.05 | 82.36 | 83.37 |
|  | Overweight | 12.02 | 13.60 | 13.64 | 13.14 |
|  | Obese | 2.95 | 3.34 | 3.99 | 3.49 |
| 20 | Not-overweight | 82.90 | 82.39 | 80.37 | 81.73 |
|  | Overweight | 14.31 | 14.67 | 15.77 | 15.00 |
|  | Obese | 2.78 | 2.93 | 3.86 | 3.26 |
| 21 | Not-overweight | 78.38 | 79.67 | 81.53 | 80.04 |
|  | Overweight | 18.05 | 15.88 | 14.39 | 15.93 |
|  | Obese | 3.56 | 4.46 | 4.09 | 4.02 |
| 22 | Not-overweight | 76.94 | 76.60 | 73.95 | 75.65 |
|  | Overweight | 17.69 | 17.97 | 21.20 | 19.17 |
|  | Obese | 5.37 | 5.44 | 4.85 | 5.18 |
| 23 | Not-overweight | 74.51 | 66.41 | 68.70 | 69.92 |
|  | Overweight | 19.61 | 25.38 | 26.03 | 23.83 |
|  | Obese | 5.88 | 8.21 | 5.27 | 6.25 |
| 24 | Not-overweight | 73.71 | 70.92 | 69.89 | 71.36 |
|  | Overweight | 18.84 | 21.75 | 22.00 | 20.94 |
|  | Obese | 7.45 | 7.33 | 8.11 | 7.69 |
| 25 | Not-overweight | 71.78 | 68.46 | 67.00 | 68.88 |
|  | Overweight | 23.11 | 23.47 | 25.83 | 24.33 |
|  | Obese | 5.11 | 8.07 | 7.17 | 6.79 |
| 26 | Not-overweight | 64.95 | 64.72 | 66.73 | 65.63 |
|  | Overweight | 26.03 | 24.72 | 25.51 | 25.45 |
|  | Obese | 9.02 | 10.56 | 7.76 | 8.92 |
| 27 | Not-overweight | 65.56 | 61.73 | 62.91 | 63.32 |
|  | Overweight | 23.69 | 27.49 | 26.18 | 25.86 |
|  | Obese | 10.74 | 10.78 | 10.91 | 10.83 |
| 28 | Not-overweight | 62.74 | 57.58 | 60.41 | 60.29 |
|  | Overweight | 28.77 | 32.58 | 31.78 | 31.14 |
|  | Obese | 8.49 | 9.83 | 7.81 | 8.58 |
| 29 | Not-overweight | 56.38 | 53.91 | 55.34 | 55.21 |
|  | Overweight | 31.91 | 35.94 | 33.12 | 33.63 |
|  | Obese | 11.70 | 10.16 | 11.54 | 11.16 |
| 30 | Not-overweight | 56.81 | 50.16 | 56.03 | 54.74 |
|  | Overweight | 32.90 | 37.78 | 31.29 | 33.53 |
|  | Obese | 10.28 | 12.06 | 12.68 | 11.74 |
| 31 | Not-overweight | 58.39 | 51.27 | 51.76 | 53.60 |
|  | Overweight | 31.54 | 36.00 | 33.49 | 33.60 |
|  | Obese | 10.07 | 12.73 | 14.75 | 12.80 |
| 32 | Not-overweight | 54.50 | 48.52 | 47.18 | 49.84 |
|  | Overweight | 33.68 | 39.35 | 37.28 | 36.71 |
|  | Obese | 11.83 | 12.13 | 15.53 | 13.45 |
| 33 | Not-overweight | 52.96 | 45.48 | 51.93 | 50.35 |
|  | Overweight | 30.84 | 37.65 | 34.83 | 34.53 |
|  | Obese | 16.20 | 16.87 | 13.24 | 15.12 |
| 34 | Not-overweight | 50.46 | 44.02 | 44.40 | 45.95 |
|  | Overweight | 37.46 | 36.44 | 40.93 | 38.68 |
|  | Obese | 12.07 | 19.53 | 14.67 | 15.37 |
| 35 | Not-overweight | 51.03 | 42.06 | 45.96 | 46.36 |
|  | Overweight | 35.40 | 40.50 | 37.87 | 37.87 |
|  | Obese | 13.57 | 17.45 | 16.17 | 15.77 |
| 36 | Not-overweight | 50.64 | 47.08 | 46.64 | 47.89 |
|  | Overweight | 33.33 | 35.38 | 37.39 | 35.67 |
|  | Obese | 16.03 | 17.54 | 15.97 | 16.44 |
| 37 | Not-overweight | 55.34 | 46.59 | 39.42 | 45.92 |
|  | Overweight | 32.69 | 34.72 | 43.98 | 38.12 |
|  | Obese | 11.97 | 18.69 | 16.60 | 15.96 |
| 38 | Not-overweight | 44.21 | 45.91 | 41.04 | 43.23 |
|  | Overweight | 37.80 | 36.16 | 40.30 | 38.49 |
|  | Obese | 17.99 | 17.92 | 18.66 | 18.27 |
| 39 | Not-overweight | 42.90 | 37.78 | 40.24 | 40.37 |
|  | Overweight | 37.29 | 43.70 | 41.42 | 40.83 |
|  | Obese | 19.80 | 18.52 | 18.34 | 18.80 |
| 40 | Not-overweight | 47.56 | 41.84 | 41.99 | 43.33 |
|  | Overweight | 35.83 | 38.87 | 37.61 | 37.51 |
|  | Obese | 16.61 | 19.29 | 20.40 | 19.16 |
| 41 | Not-overweight | 43.80 | 38.75 | 35.09 | 38.38 |
|  | Overweight | 37.98 | 41.70 | 45.61 | 42.54 |
|  | Obese | 18.22 | 19.56 | 19.30 | 19.09 |
| 42 | Not-overweight | 50.76 | 37.06 | 33.58 | 39.34 |
|  | Overweight | 35.35 | 43.13 | 45.40 | 41.97 |
|  | Obese | 13.90 | 19.81 | 21.01 | 18.69 |
| 43 | Not-overweight | 47.29 | 45.35 | 35.81 | 41.38 |
|  | Overweight | 35.66 | 35.66 | 43.67 | 39.43 |
|  | Obese | 17.05 | 18.99 | 20.52 | 19.20 |
| 44 | Not-overweight | 43.41 | 34.63 | 37.39 | 38.26 |
|  | Overweight | 39.92 | 42.41 | 41.59 | 41.37 |
|  | Obese | 16.67 | 22.96 | 21.02 | 20.37 |
| 45 | Not-overweight | 41.64 | 34.48 | 31.25 | 34.79 |
|  | Overweight | 39.41 | 46.55 | 44.76 | 43.89 |
|  | Obese | 18.96 | 18.97 | 23.99 | 21.33 |
| 46 | Not-overweight | 40.81 | 36.18 | 29.80 | 34.16 |
|  | Overweight | 43.95 | 42.28 | 44.81 | 43.93 |
|  | Obese | 15.25 | 21.54 | 25.39 | 21.91 |
| 47 | Not-overweight | 43.75 | 30.96 | 30.51 | 33.84 |
|  | Overweight | 36.25 | 43.77 | 45.76 | 42.90 |
|  | Obese | 20.00 | 25.27 | 23.73 | 23.26 |
| 48 | Not-overweight | 41.18 | 28.44 | 34.77 | 34.88 |
|  | Overweight | 38.66 | 41.78 | 41.77 | 40.99 |
|  | Obese | 20.17 | 29.78 | 23.46 | 24.13 |
| 49 | Not-overweight | 38.04 | 33.62 | 21.85 | 28.51 |
|  | Overweight | 41.30 | 37.87 | 49.10 | 44.38 |
|  | Obese | 20.65 | 28.51 | 29.05 | 27.11 |
| 50 | Not-overweight | 37.23 | 32.08 | 29.57 | 32.12 |
|  | Overweight | 42.42 | 38.75 | 43.91 | 42.21 |
|  | Obese | 20.35 | 29.17 | 26.52 | 25.67 |
| 51 | Not-overweight | 49.35 | 33.76 | 25.52 | 33.18 |
|  | Overweight | 29.87 | 42.04 | 48.66 | 42.59 |
|  | Obese | 20.78 | 24.20 | 25.82 | 24.23 |
| 52 | Not-overweight | 43.84 | 30.94 | 27.43 | 32.55 |
|  | Overweight | 36.53 | 44.84 | 44.90 | 42.74 |
|  | Obese | 19.63 | 24.22 | 27.67 | 24.71 |
| 53 | Not-overweight | 50.26 | 32.56 | 26.92 | 34.47 |
|  | Overweight | 33.85 | 37.79 | 47.53 | 41.59 |
|  | Obese | 15.90 | 29.65 | 25.55 | 23.94 |
| 54 | Not-overweight | 45.21 | 39.18 | 24.27 | 33.33 |
|  | Overweight | 35.16 | 37.11 | 43.69 | 39.88 |
|  | Obese | 19.63 | 23.71 | 32.04 | 26.79 |
| 55 | Not-overweight | 52.27 | 36.46 | 27.17 | 35.71 |
|  | Overweight | 28.98 | 35.91 | 43.70 | 38.10 |
|  | Obese | 18.75 | 27.62 | 29.13 | 26.19 |
| 56 | Not-overweight | 43.55 | 33.54 | 24.01 | 31.66 |
|  | Overweight | 33.33 | 39.02 | 49.24 | 42.42 |
|  | Obese | 23.12 | 27.44 | 26.75 | 25.92 |
| 57 | Not-overweight | 39.39 | 32.28 | 28.70 | 31.90 |
|  | Overweight | 35.61 | 37.80 | 40.12 | 38.59 |
|  | Obese | 25.00 | 29.92 | 31.17 | 29.50 |
| 58 | Not-overweight | 43.80 | 27.78 | 24.91 | 29.85 |
|  | Overweight | 38.84 | 45.14 | 45.55 | 43.96 |
|  | Obese | 17.36 | 27.08 | 29.54 | 26.19 |
| 59 | Not-overweight | 48.72 | 31.07 | 29.22 | 34.56 |
|  | Overweight | 32.48 | 45.63 | 39.51 | 39.09 |
|  | Obese | 18.80 | 23.30 | 31.28 | 26.35 |
| 60 | Not-overweight | 44.90 | 36.88 | 26.73 | 33.67 |
|  | Overweight | 41.50 | 42.55 | 42.57 | 42.30 |
|  | Obese | 13.61 | 20.57 | 30.69 | 24.03 |
| 61 | Not-overweight | 51.35 | 40.54 | 30.91 | 38.02 |
|  | Overweight | 28.38 | 43.24 | 42.42 | 39.30 |
|  | Obese | 20.27 | 16.22 | 26.67 | 22.68 |
| 62 | Not-overweight | 40.31 | 35.29 | 23.29 | 30.58 |
|  | Overweight | 40.31 | 46.22 | 52.61 | 47.89 |
|  | Obese | 19.38 | 18.49 | 24.10 | 21.53 |
| 63 | Not-overweight | 43.44 | 46.32 | 27.98 | 37.18 |
|  | Overweight | 38.52 | 36.76 | 46.33 | 41.60 |
|  | Obese | 18.03 | 16.91 | 25.69 | 21.22 |
| 64 | Not-overweight | 50.65 | 31.43 | 26.34 | 34.94 |
|  | Overweight | 35.06 | 44.29 | 41.07 | 40.15 |
|  | Obese | 14.29 | 24.29 | 32.59 | 24.90 |

†SES = Socioeconomic status; The World Bank’s Wealth Index (WI)[1] was used as SES indicator; Lower SES= the lowest and second quintiles of WI; Middle SES= middle quintile of WI; Higher SES= fourth and highest quintiles of WI.

**Table 2. Prevalence rates by BMI category, age, and socioeconomic status in 2010 ^†^**

| **Age** | **Nutritional Status** | **Prevalence rates** | | | |
| --- | --- | --- | --- | --- | --- |
|  |  | **Lower SES** | **Middle SES** | **Higher SES** | **Complete Urban Population** |
| 0 | Not-overweight | 76.49 | 79.15 | 73.68 | 76.48 |
|  | Overweight | 17.87 | 16.29 | 20.17 | 18.04 |
|  | Obese | 5.64 | 4.56 | 6.15 | 5.47 |
| 1 | Not-overweight | 70.66 | 70.70 | 69.39 | 70.31 |
|  | Overweight | 24.35 | 23.89 | 23.08 | 23.85 |
|  | Obese | 4.99 | 5.41 | 7.54 | 5.84 |
| 2 | Not-overweight | 73.01 | 73.63 | 71.95 | 72.88 |
|  | Overweight | 22.81 | 20.59 | 21.65 | 21.85 |
|  | Obese | 4.18 | 5.77 | 6.40 | 5.27 |
| 3 | Not-overweight | 75.70 | 76.22 | 75.38 | 75.76 |
|  | Overweight | 19.13 | 18.05 | 18.24 | 18.55 |
|  | Obese | 5.17 | 5.73 | 6.38 | 5.68 |
| 4 | Not-overweight | 80.69 | 80.64 | 76.30 | 79.36 |
|  | Overweight | 15.83 | 16.04 | 17.11 | 16.28 |
|  | Obese | 3.47 | 3.32 | 6.59 | 4.36 |
| 5 | Not-overweight | 83.61 | 80.89 | 74.82 | 80.25 |
|  | Overweight | 12.59 | 14.01 | 17.40 | 14.41 |
|  | Obese | 3.80 | 5.10 | 7.78 | 5.34 |
| 6 | Not-overweight | 84.58 | 80.57 | 72.00 | 79.65 |
|  | Overweight | 12.03 | 12.43 | 17.07 | 13.66 |
|  | Obese | 3.39 | 7.00 | 10.93 | 6.69 |
| 7 | Not-overweight | 82.47 | 78.29 | 72.65 | 78.26 |
|  | Overweight | 11.85 | 15.27 | 17.83 | 14.67 |
|  | Obese | 5.67 | 6.44 | 9.52 | 7.07 |
| 8 | Not-overweight | 82.68 | 76.20 | 68.55 | 76.30 |
|  | Overweight | 12.41 | 16.35 | 19.04 | 15.65 |
|  | Obese | 4.90 | 7.45 | 12.41 | 8.05 |
| 9 | Not-overweight | 80.86 | 75.15 | 67.28 | 75.10 |
|  | Overweight | 14.06 | 17.73 | 20.98 | 17.20 |
|  | Obese | 5.08 | 7.12 | 11.74 | 7.70 |
| 10 | Not-overweight | 81.53 | 75.11 | 65.88 | 74.71 |
|  | Overweight | 13.90 | 18.81 | 21.38 | 17.68 |
|  | Obese | 4.57 | 6.08 | 12.73 | 7.61 |
| 11 | Not-overweight | 81.39 | 77.54 | 69.43 | 76.35 |
|  | Overweight | 13.12 | 16.69 | 20.54 | 16.55 |
|  | Obese | 5.48 | 5.77 | 10.04 | 7.09 |
| 12 | Not-overweight | 82.31 | 77.08 | 71.06 | 76.90 |
|  | Overweight | 13.57 | 17.93 | 19.95 | 17.07 |
|  | Obese | 4.12 | 4.99 | 8.98 | 6.03 |
| 13 | Not-overweight | 82.75 | 80.34 | 75.24 | 79.44 |
|  | Overweight | 13.96 | 15.03 | 19.87 | 16.34 |
|  | Obese | 3.29 | 4.63 | 4.89 | 4.22 |
| 14 | Not-overweight | 85.64 | 82.51 | 81.05 | 83.13 |
|  | Overweight | 11.73 | 14.19 | 15.45 | 13.74 |
|  | Obese | 2.63 | 3.31 | 3.50 | 3.13 |
| 15 | Not-overweight | 83.01 | 84.47 | 80.99 | 82.69 |
|  | Overweight | 13.68 | 12.82 | 14.73 | 13.81 |
|  | Obese | 3.31 | 2.71 | 4.29 | 3.49 |
| 16 | Not-overweight | 84.50 | 85.44 | 80.92 | 83.38 |
|  | Overweight | 12.97 | 12.33 | 15.85 | 13.90 |
|  | Obese | 2.53 | 2.23 | 3.23 | 2.72 |
| 17 | Not-overweight | 82.99 | 82.24 | 81.29 | 82.17 |
|  | Overweight | 12.67 | 12.83 | 14.01 | 13.21 |
|  | Obese | 4.34 | 4.93 | 4.71 | 4.63 |
| 18 | Not-overweight | 83.03 | 81.42 | 80.83 | 81.80 |
|  | Overweight | 14.42 | 13.25 | 15.04 | 14.32 |
|  | Obese | 2.55 | 5.33 | 4.14 | 3.88 |
| 19 | Not-overweight | 81.44 | 79.96 | 78.72 | 79.96 |
|  | Overweight | 14.53 | 15.77 | 14.51 | 14.85 |
|  | Obese | 4.03 | 4.27 | 6.77 | 5.19 |
| 20 | Not-overweight | 76.47 | 77.98 | 75.95 | 76.71 |
|  | Overweight | 16.03 | 15.40 | 19.72 | 17.25 |
|  | Obese | 7.50 | 6.62 | 4.33 | 6.04 |
| 21 | Not-overweight | 78.02 | 76.62 | 74.50 | 76.31 |
|  | Overweight | 16.25 | 18.47 | 18.30 | 17.63 |
|  | Obese | 5.73 | 4.91 | 7.20 | 6.06 |
| 22 | Not-overweight | 68.87 | 71.61 | 69.42 | 69.88 |
|  | Overweight | 22.24 | 22.50 | 21.71 | 22.13 |
|  | Obese | 8.90 | 5.89 | 8.87 | 7.98 |
| 23 | Not-overweight | 68.44 | 65.67 | 67.72 | 67.42 |
|  | Overweight | 25.25 | 26.18 | 24.17 | 25.09 |
|  | Obese | 6.31 | 8.15 | 8.11 | 7.50 |
| 24 | Not-overweight | 62.79 | 66.05 | 64.70 | 64.42 |
|  | Overweight | 26.08 | 24.13 | 28.30 | 26.36 |
|  | Obese | 11.13 | 9.82 | 7.00 | 9.23 |
| 25 | Not-overweight | 59.34 | 60.88 | 62.46 | 60.91 |
|  | Overweight | 29.92 | 27.54 | 27.64 | 28.41 |
|  | Obese | 10.74 | 11.58 | 9.90 | 10.68 |
| 26 | Not-overweight | 55.27 | 57.86 | 59.29 | 57.48 |
|  | Overweight | 30.27 | 30.61 | 30.69 | 30.52 |
|  | Obese | 14.46 | 11.53 | 10.02 | 12.00 |
| 27 | Not-overweight | 54.48 | 55.93 | 55.52 | 55.30 |
|  | Overweight | 31.53 | 32.02 | 31.68 | 31.73 |
|  | Obese | 13.99 | 12.06 | 12.80 | 12.97 |
| 28 | Not-overweight | 51.96 | 53.67 | 50.74 | 51.97 |
|  | Overweight | 34.64 | 32.24 | 35.65 | 34.35 |
|  | Obese | 13.41 | 14.08 | 13.61 | 13.68 |
| 29 | Not-overweight | 48.86 | 54.56 | 49.26 | 50.66 |
|  | Overweight | 35.68 | 31.54 | 36.53 | 34.78 |
|  | Obese | 15.47 | 13.90 | 14.21 | 14.55 |
| 30 | Not-overweight | 46.79 | 44.38 | 47.14 | 46.24 |
|  | Overweight | 35.36 | 40.12 | 37.43 | 37.53 |
|  | Obese | 17.85 | 15.50 | 15.43 | 16.23 |
| 31 | Not-overweight | 46.80 | 50.46 | 48.14 | 48.44 |
|  | Overweight | 37.44 | 34.03 | 38.66 | 36.86 |
|  | Obese | 15.75 | 15.51 | 13.20 | 14.70 |
| 32 | Not-overweight | 46.64 | 43.05 | 43.87 | 44.53 |
|  | Overweight | 32.99 | 37.36 | 39.03 | 36.58 |
|  | Obese | 20.37 | 19.59 | 17.10 | 18.89 |
| 33 | Not-overweight | 42.77 | 44.56 | 42.57 | 43.26 |
|  | Overweight | 39.26 | 39.02 | 38.41 | 38.87 |
|  | Obese | 17.98 | 16.42 | 19.02 | 17.87 |
| 34 | Not-overweight | 41.92 | 44.27 | 43.21 | 43.04 |
|  | Overweight | 37.50 | 35.11 | 39.06 | 37.42 |
|  | Obese | 20.58 | 20.61 | 17.74 | 19.54 |
| 35 | Not-overweight | 39.92 | 42.82 | 43.08 | 41.89 |
|  | Overweight | 38.72 | 36.14 | 39.42 | 38.25 |
|  | Obese | 21.36 | 21.04 | 17.50 | 19.86 |
| 36 | Not-overweight | 42.17 | 39.42 | 38.72 | 40.02 |
|  | Overweight | 38.80 | 40.21 | 43.97 | 41.24 |
|  | Obese | 19.04 | 20.37 | 17.32 | 18.75 |
| 37 | Not-overweight | 46.78 | 34.30 | 37.66 | 39.48 |
|  | Overweight | 36.59 | 46.19 | 45.64 | 42.97 |
|  | Obese | 16.63 | 19.51 | 16.70 | 17.55 |
| 38 | Not-overweight | 37.98 | 39.66 | 35.28 | 37.31 |
|  | Overweight | 44.04 | 39.18 | 45.41 | 43.27 |
|  | Obese | 17.98 | 21.15 | 19.30 | 19.42 |
| 39 | Not-overweight | 38.16 | 38.82 | 37.91 | 38.24 |
|  | Overweight | 35.75 | 41.28 | 41.18 | 39.64 |
|  | Obese | 26.09 | 19.90 | 20.92 | 22.12 |
| 40 | Not-overweight | 36.65 | 35.15 | 33.67 | 34.99 |
|  | Overweight | 40.24 | 41.95 | 44.90 | 42.66 |
|  | Obese | 23.11 | 22.90 | 21.43 | 22.34 |
| 41 | Not-overweight | 35.84 | 35.50 | 35.73 | 35.70 |
|  | Overweight | 38.44 | 39.05 | 43.76 | 40.98 |
|  | Obese | 25.71 | 25.44 | 20.51 | 23.32 |
| 42 | Not-overweight | 37.79 | 36.72 | 33.38 | 35.57 |
|  | Overweight | 40.50 | 43.42 | 47.18 | 44.20 |
|  | Obese | 21.71 | 19.86 | 19.44 | 20.22 |
| 43 | Not-overweight | 33.68 | 39.63 | 34.11 | 35.52 |
|  | Overweight | 42.63 | 36.17 | 43.48 | 41.21 |
|  | Obese | 23.68 | 24.20 | 22.41 | 23.26 |
| 44 | Not-overweight | 36.22 | 33.16 | 34.95 | 34.87 |
|  | Overweight | 41.11 | 39.12 | 43.81 | 41.70 |
|  | Obese | 22.67 | 27.72 | 21.24 | 23.43 |
| 45 | Not-overweight | 34.36 | 37.37 | 29.61 | 33.15 |
|  | Overweight | 41.23 | 38.13 | 47.90 | 43.25 |
|  | Obese | 24.41 | 24.49 | 22.49 | 23.61 |
| 46 | Not-overweight | 40.56 | 30.55 | 32.24 | 34.09 |
|  | Overweight | 38.78 | 43.60 | 45.33 | 43.05 |
|  | Obese | 20.66 | 25.85 | 22.43 | 22.87 |
| 47 | Not-overweight | 35.68 | 30.62 | 30.60 | 32.02 |
|  | Overweight | 39.73 | 48.60 | 46.66 | 45.24 |
|  | Obese | 24.59 | 20.79 | 22.74 | 22.73 |
| 48 | Not-overweight | 35.41 | 29.75 | 28.18 | 30.46 |
|  | Overweight | 38.24 | 44.63 | 47.01 | 44.12 |
|  | Obese | 26.35 | 25.62 | 24.81 | 25.42 |
| 49 | Not-overweight | 34.97 | 28.84 | 30.04 | 31.14 |
|  | Overweight | 38.15 | 44.20 | 44.40 | 42.55 |
|  | Obese | 26.88 | 26.96 | 25.56 | 26.31 |
| 50 | Not-overweight | 34.25 | 28.48 | 26.64 | 29.25 |
|  | Overweight | 42.19 | 42.07 | 47.04 | 44.46 |
|  | Obese | 23.56 | 29.45 | 26.32 | 26.29 |
| 51 | Not-overweight | 34.08 | 28.05 | 26.31 | 28.97 |
|  | Overweight | 41.40 | 41.58 | 48.39 | 44.57 |
|  | Obese | 24.52 | 30.36 | 25.30 | 26.46 |
| 52 | Not-overweight | 35.44 | 34.02 | 24.87 | 29.97 |
|  | Overweight | 40.51 | 37.11 | 46.94 | 42.78 |
|  | Obese | 24.05 | 28.87 | 28.20 | 27.25 |
| 53 | Not-overweight | 32.53 | 31.50 | 27.73 | 29.99 |
|  | Overweight | 43.15 | 42.12 | 42.97 | 42.80 |
|  | Obese | 24.32 | 26.37 | 29.30 | 27.21 |
| 54 | Not-overweight | 39.49 | 29.22 | 25.00 | 30.33 |
|  | Overweight | 33.44 | 39.92 | 45.29 | 40.48 |
|  | Obese | 27.07 | 30.86 | 29.71 | 29.19 |
| 55 | Not-overweight | 36.00 | 33.87 | 25.62 | 30.44 |
|  | Overweight | 35.33 | 36.69 | 43.40 | 39.59 |
|  | Obese | 28.67 | 29.44 | 30.98 | 29.97 |
| 56 | Not-overweight | 36.44 | 27.62 | 23.06 | 27.95 |
|  | Overweight | 40.08 | 45.19 | 43.69 | 43.10 |
|  | Obese | 23.48 | 27.20 | 33.25 | 28.95 |
| 57 | Not-overweight | 37.25 | 28.78 | 30.22 | 31.55 |
|  | Overweight | 37.75 | 42.93 | 42.00 | 41.21 |
|  | Obese | 25.00 | 28.29 | 27.78 | 27.24 |
| 58 | Not-overweight | 39.50 | 29.03 | 25.58 | 29.73 |
|  | Overweight | 42.00 | 40.09 | 47.47 | 44.30 |
|  | Obese | 18.50 | 30.88 | 26.96 | 25.97 |
| 59 | Not-overweight | 36.23 | 34.78 | 23.80 | 29.64 |
|  | Overweight | 42.03 | 30.98 | 45.57 | 41.22 |
|  | Obese | 21.74 | 34.24 | 30.63 | 29.13 |
| 60 | Not-overweight | 41.35 | 30.54 | 24.11 | 30.58 |
|  | Overweight | 37.55 | 44.33 | 45.94 | 43.17 |
|  | Obese | 21.10 | 25.12 | 29.95 | 26.26 |
| 61 | Not-overweight | 38.75 | 27.59 | 25.86 | 29.55 |
|  | Overweight | 31.88 | 42.07 | 47.35 | 42.17 |
|  | Obese | 29.38 | 30.34 | 26.79 | 28.27 |
| 62 | Not-overweight | 40.22 | 29.95 | 22.81 | 29.11 |
|  | Overweight | 41.90 | 42.64 | 47.95 | 44.99 |
|  | Obese | 17.88 | 27.41 | 29.24 | 25.91 |
| 63 | Not-overweight | 37.80 | 31.65 | 28.35 | 31.54 |
|  | Overweight | 35.37 | 43.67 | 39.94 | 39.69 |
|  | Obese | 26.83 | 24.68 | 31.71 | 28.77 |
| 64 | Not-overweight | 43.65 | 31.21 | 28.91 | 33.77 |
|  | Overweight | 36.46 | 41.84 | 42.52 | 40.58 |
|  | Obese | 19.89 | 26.95 | 28.57 | 25.65 |

†SES = Socioeconomic status; The World Bank’s Wealth Index (WI)[1] was used as SES indicator; Lower SES= the lowest and second quintiles of WI; Middle SES= middle quintile of WI; Higher SES= fourth and highest quintiles of WI.

**Section 2. Data and model parameters used in the system dynamics models**

The following tables describe the data and parameters used in the two system dynamics (SD) models: SD model of the entire urban population and SD model segregated by socioeconomic status (SES).

**Table 3. Mortality rates and population size by age group without segregate by socioeconomic status^†^**

| **Age group** | **Mortality rates (R) (%/year)** | **Not- overweight population in 2005 (Number of people)^1^** | **Overweight population in 2005 (Number of people)^2^** | **Obese population in 2005**  **(Number of people)^3^** |
| --- | --- | --- | --- | --- |
| **0-4** | 0.428 | 2317709 | 541834 | 146136 |
| **5-9** | 0.033 | 2565461 | 425742 | 146196 |
| **10-14** | 0.034 | 2652272 | 437248 | 127990 |
| **15-19** | 0.109 | 2604610 | 388481 | 84750 |
| **20-24** | 0.177 | 2159468 | 541105 | 150900 |
| **25-29** | 0.184 | 1624490 | 720939 | 236880 |
| **30-34** | 0.192 | 1164064 | 813016 | 314075 |
| **35-39** | 0.207 | 1036627 | 883929 | 394400 |
| **40-44** | 0.243 | 884598 | 889124 | 423373 |
| **45-49** | 0.326 | 610808 | 791179 | 429289 |
| **50-54** | 0.502 | 480614 | 607417 | 365470 |
| **55-59** | 0.732 | 368799 | 454312 | 301368 |

**^†^**Values used in this table are for the urban population in Colombia

^1^ Initial value used in the stock of not-overweight population in 2005.

^2^ Initial value used in the stock of overweight population in 2005.

^3^ Initial value used in the stock of obese population in 2005.

**Table 4. Mortality rates and population size by age group for the lower socioeconomic status population^†^**

| **Age group** | **Mortality rates (R) (%/year)** | **Not- overweight population in 2005 (Number of people)^1^** | **Overweight population in 2005 (Number of people)^2^** | **Obese population in 2005**  **(Number of people)^3^** |
| --- | --- | --- | --- | --- |
| **0-4** | 0.428 | 990480 | 209674 | 45535 |
| **5-9** | 0.033 | 1115737 | 141302 | 28750 |
| **10-14** | 0.034 | 1102306 | 145137 | 25720 |
| **15-19** | 0.109 | 940671 | 127914 | 21056 |
| **20-24** | 0.177 | 698102 | 159283 | 45346 |
| **25-29** | 0.184 | 496735 | 204398 | 68133 |
| **30-34** | 0.192 | 373709 | 227804 | 82296 |
| **35-39** | 0.207 | 317235 | 229454 | 102887 |
| **40-44** | 0.243 | 271962 | 213949 | 95043 |
| **45-49** | 0.326 | 182285 | 175775 | 83866 |
| **50-54** | 0.502 | 165792 | 133726 | 71418 |
| **55-59** | 0.732 | 126198 | 92294 | 57260 |

**^†^**Values used in this table are for the urban population

^1^ Initial value used in the stock of not-overweight population in 2005 for lower SES.

^2^ Initial value used in the stock of overweight population in 2005 for lower SES.

^3^ Initial value used in the stock of obese population in 2005 for lower SES.

**Table 5. Mortality rates and population size by age group for the middle socioeconomic status population^†^**

| **Age group** | **Mortality rates (R) (%/year)** | **Not- overweight population in 2005 (Number of people)^1^** | **Overweight population in 2005 (Number of people)^2^** | **Obese population in 2005**  **(Number of people)^3^** |
| --- | --- | --- | --- | --- |
| **0-4** | 0.428 | 647377 | 157785 | 43417 |
| **5-9** | 0.033 | 703453 | 107047 | 43431 |
| **10-14** | 0.034 | 746506 | 125540 | 41643 |
| **15-19** | 0.109 | 681684 | 98436 | 27373 |
| **20-24** | 0.177 | 584546 | 148232 | 43822 |
| **25-29** | 0.184 | 457123 | 214301 | 73282 |
| **30-34** | 0.192 | 304136 | 238935 | 94222 |
| **35-39** | 0.207 | 282939 | 242927 | 115544 |
| **40-44** | 0.243 | 233698 | 238636 | 118495 |
| **45-49** | 0.326 | 160457 | 208709 | 119864 |
| **50-54** | 0.502 | 120609 | 144293 | 94374 |
| **55-59** | 0.732 | 88150 | 108869 | 73835 |

**^†^**Values used in this table are for the urban population

^1^ Initial value used in the stock of not-overweight population in 2005 for middle SES.

^2^ Initial value used in the stock of overweight population in 2005 for middle SES.

^3^ Initial value used in the stock of obese population in 2005 for middle SES.

**Table 6. Mortality rates and population size by age group for the higher socioeconomic status population^†^**

| **Age group** | **Mortality rates (R) (%/year)** | **Not- overweight population in 2005 (Number of people)^1^** | **Overweight population in 2005 (Number of people)^2^** | **Obese population in 2005**  **(Number of people)^3^** |
| --- | --- | --- | --- | --- |
| **0-4** | 0.428 | 679852 | 174375 | 57184 |
| **5-9** | 0.033 | 746272 | 177392 | 74015 |
| **10-14** | 0.034 | 803459 | 166571 | 60627 |
| **15-19** | 0.109 | 982256 | 162130 | 36321 |
| **20-24** | 0.177 | 876819 | 233590 | 61732 |
| **25-29** | 0.184 | 670632 | 302240 | 95465 |
| **30-34** | 0.192 | 486219 | 346277 | 137557 |
| **35-39** | 0.207 | 436453 | 411548 | 175969 |
| **40-44** | 0.243 | 378937 | 436539 | 209835 |
| **45-49** | 0.326 | 268066 | 406695 | 225559 |
| **50-54** | 0.502 | 194213 | 329397 | 199679 |
| **55-59** | 0.732 | 154451 | 253149 | 170273 |

**^†^**Values used in this table are for the urban population

^1^ Initial value used in the stock of not-overweight population in 2005 for higher SES.

^2^ Initial value used in the stock of overweight population in 2005 for higher SES.

^3^ Initial value used in the stock of obese population in 2005 for higher SES.

**Table 7. Fraction of births by body mass index category and socioeconomic status ^†^**

| **Population group** | **Lower SES (%/year)** | **Middle SES (%/year)** | **Higher SES (%/year)** | **Complete Urban Population (%/year)** |
| --- | --- | --- | --- | --- |
| **Not- overweight** | 79.048 | 84.874 | 76.577 | 79.82 |
| **Overweight** | 15.238 | 10.084 | 18.018 | 14.68 |
| **Obese** | 5.714 | 5.042 | 5.405 | 5.50 |

**^†^** The fractions of births by each BMI category correspond to the prevalences by BMI category of children aged 0 to 2 months

**Table 8. Data and projections of the Fertility rates^†^**

| Year | Fertility rate | Year | Fertility rate | Year | Fertility rate |
| --- | --- | --- | --- | --- | --- |
| 1960 | 6.81 | 1985 | 3.43 | 2010 | 2.38 |
| 1961 | 6.8 | 1986 | 3.35 | 2011 | 2.35 |
| 1962 | 6.78 | 1987 | 3.28 | 2012 | 2.32 |
| 1963 | 6.73 | 1988 | 3.21 | 2013 | 2.29 |
| 1964 | 6.66 | 1989 | 3.16 | 2014 | 2.26 |
| 1965 | 6.56 | 1990 | 3.1 | 2015 | 2.23 |
| 1966 | 6.42 | 1991 | 3.06 | 2016 | 2.2 |
| 1967 | 6.25 | 1992 | 3.01 | 2017 | 2.17 |
| 1968 | 6.05 | 1993 | 2.97 | 2018 | 2.14 |
| 1969 | 5.83 | 1994 | 2.92 | 2019 | 2.11 |
| 1970 | 5.6 | 1995 | 2.87 | 2020 | 2.08 |
| 1971 | 5.37 | 1996 | 2.82 | 2021 | 2.05 |
| 1972 | 5.15 | 1997 | 2.78 | 2022 | 2.02 |
| 1973 | 4.96 | 1998 | 2.73 | 2023 | 1.99 |
| 1974 | 4.78 | 1999 | 2.68 | 2024 | 1.96 |
| 1975 | 4.63 | 2000 | 2.64 | 2025 | 1.93 |
| 1976 | 4.5 | 2001 | 2.6 | 2026 | 1.9 |
| 1977 | 4.37 | 2002 | 2.57 | 2027 | 1.87 |
| 1978 | 4.25 | 2003 | 2.54 | 2028 | 1.84 |
| 1979 | 4.12 | 2004 | 2.52 | 2029 | 1.81 |
| 1980 | 3.99 | 2005 | 2.5 | 2030 | 1.78 |
| 1981 | 3.87 | 2006 | 2.48 |  |  |
| 1982 | 3.74 | 2007 | 2.45 |  |  |
| 1983 | 3.63 | 2008 | 2.43 |  |  |
| 1984 | 3.53 | 2009 | 2.41 |  |  |

**^†^** [Data of Fertility rate of world Data Bank](http://databank.worldbank.org/data/home.aspx)(1960–2014)[2] was used to forecast the fertility rate from 2015 to 2030. Holt-Winters no seasonal method in EViews 5 (Quantitative Micro Software, LLC) was used to forecast the fertility rate. In the SD model we used data from 2005 to 2030.

**References**

1. Rutstein SO, Jonhson K. The DHS Wealth Index. DHS Comparative Reports no. 6. Calverton, MA: ORC Macro; 2004.

2. World Data Bank. World Development Indicators [Internet]. [cited 8 Dec 2016]. Available: http://databank.worldbank.org/data/reports.aspx?source=World%20Development%20Indicators
