# Supplementary material for: Towards a novel model for studying the nutritional stage dynamics of the Colombian population by age and socioeconomic status
Source: PLoS One. 2018 Feb 8;13(2):e0191929. doi: 10.1371/journal.pone.0191929 (PMC5805245; doi:10.1371/journal.pone.0191929)
Supplement: S3 Appendix — This file describes the system dynamics model stratified by socioeconomic status groups. (DOCX) [file pone.0191929.s003.docx]

**Supplementary Information 3**

**Supplement to: Towards a novel model for studying the nutritional stage dynamics of the Colombian population by age and socioeconomic**

**Table of contents**

**Section 1. Model of nutritional stage dynamics by BMI and SES** 3

**Section 2. Heuristic to estimate the transference rates between BMI categories by age and SES** 6

**References** 8

**Section 1. Model of nutritional stage dynamics by BMI and SES**

To study the nutritional stage dynamics by body mass index (BMI) category and socioeconomic status (SES), we used the SD model proposed in the main article, but stratified by SES groups. We used the World Bank’s Wealth Index (WI) [1] as our SES indicator. It allows categorization of the population into socio-economic groups, captures quality of life markers, and is well validated. We grouped the WI categories into three socio-economic groups: the lowest and second quintiles, the middle quintile, and the fourth and highest quintiles. The population-level SD model includes ageing chains for three BMI categories (*not overweight*, *overweight*, and *obese*) for each SES group; thus, the population aged 0-59 years was divided into intervals of 5 year. The population ageing structure also included births and deaths for each cohort, with mortality rates differed among the age groups [2,3]. Given the purpose of the study and aims defined for the proposed SD model, we assumed the same mortality rates for each BMI category, but differed across age groups, and that the net migration was zero. We made this assumption because there are no data for Colombia regarding mortality rates or net migration patterns by BMI category and SES group. Another reason we assumed that the net migration was zero, was because the proportion of individuals who migrate from Colombia is less than 0.5% per year[4].

We classified individuals into the three BMI categories based on the following WHO criteria: the 2006 WHO child growth standards for children under 5 [5], the 2006 WHO child growth references for children aged 5 to 17 years [6], and the WHO cut-off points for aged at least 18 years [7]. For children and adolescents aged 0–17 years, the WHO system defines *not overweight* as a BMI for age and sex *z*-score ≤1 standard deviation, *overweight* as a BMI for age and sex *z*-score >1 standard deviation and ≤ 2 standard deviations, and *obese* as a BMI for age and sex *z*-score >2 standard deviations. For adults, WHO defines *not overweight* as a BMI <25 kg/m^2^, *overweight* as a BMI ≥25 and <30 kg/m^2^, and *obese* as a BMI ≥30 kg/m^2^.

The mathematical form of the model by SES group is shown below (Equations (1-3) correspond to the net rate of change of the population in each BMI category for the age group of 0 to four years by SES group. Equations (4-6) correspond to the net rate of change of the population in each BMI category for the rest of the age groups by SES group):

$$\frac{dN_{0,k}(t)}{dt}=B_{N,k}(t)+{\tau_{4,0,k}W}_{0,k}(t)-{\tau_{1,0,k}N}_{0,k}(t)-E_{0,k}^{N}(t)\left( 1-S_{0,k} \right)-E_{0,k}^{N}(t)S_{0,k} (1)$$

$$\frac{dW_{0,k}\left( t \right)}{dt}=B_{W,k}\left( t \right)+{\tau_{1,0,k}N}_{0,k}\left( t \right)+{\tau_{3,0,k}O}_{0,k}\left( t \right)-W_{0,k}\left( t \right)\left( \tau_{2,0,k}+\tau_{4,0,k} \right)-E_{0,k}^{W}\left( t \right)\left( 1-S_{0,k} \right)-E_{0,k}^{W}\left( t \right)S_{0,k} (2)$$

$$\frac{dO_{0,k}(t)}{dt}=B_{O,k}(t)+{\tau_{2,0,k}W}_{0,k}(t)-{\tau_{3,0,k}O}_{0,k}(t)-E_{0,k}^{O}(t)\left( 1-S_{0,k} \right)-E_{0,k}^{O}\left( t \right)S_{0,k} (3)$$

$$\frac{dN_{i,k}\left( t \right)}{dt}={E_{i-1,k}^{N}\left( t \right)S_{i-1,k}+\tau_{4,i,k}W}_{i,k}\left( t \right)-{\tau_{1,i,k}N}_{i,k}\left( t \right)-E_{i,k}^{N}\left( t \right)\left( 1-S_{i,k} \right)-E_{i,k}^{N}\left( t \right)S_{i,k}, for i\in\left( 1,\ldots,n-1 \right) (4)$$

$$\frac{dW_{i,k}\left( t \right)}{dt}=E_{i-1,k}^{W}\left( t \right)S_{i-1,k}+{\tau_{1,i,k}N}_{i,k}\left( t \right)+{\tau_{3,i,k}O}_{i}\left( t \right)-W_{i,k}\left( t \right)\left( \tau_{2,i,k}+\tau_{4,i,k} \right)-E_{i,k}^{W}\left( t \right)\left( 1-S_{i,k} \right)$$

$-E_{i,k}^{W}{\left( t \right)S}_{i,k}, for i\in\left( 1,\ldots,n-1 \right)$ $(5)$

$$\frac{dO_{i,k}(t)}{dt}=E_{i-1,k}^{O}\left( t \right)S_{i-1,k}+{\tau_{2,i,k}W}_{i,k}\left( t \right)-{\tau_{3,i,k}O}_{i,k}\left( t \right)-E_{i,k}^{O}\left( t \right)\left( 1-S_{i,k} \right)-E_{i,k}^{O}\left( t \right)S_{i,k}, for i\in\left( 1,\ldots,n-1 \right) (6)$$

where *i* ∈ (0,….,11) represents the age groups, in intervals of 5 year ds, of the simulated population (0–4, 5–9,...,55–59); *k* ∈ (1, 2, 3) represents the SES groups (lower, middle, and higher SES); $n$ corresponds to the total number of age groups (12); *N_i,k_(t), W_i,k_(t),* and *O_i,k_(t)* are the populations of *not-overweight*, *overweight*, and *obese* individuals in the age group *i* and SES group *k*, respectively, at time *t* (unit: people); *B_N,k_(t), B_W,k_(t),* and *B_O,k_(t)* are the births for each BMI category and SES group of the first age group at time *t* (unit: people per year); and *E_i,k_^N^(t), E_i,k_^W^(t),* and *E_i,k_^O^(t)* are the exit rates of individuals per year for each age group *i,* SES group *k*, and BMI category at time $t$ (unit: people per year). An exit rate is the total number of individuals per year that leave each age group by BMI category. There are two groups of individuals to whom the exit rate is applied: those who mature into the next age group (e.g., *E_i,k_^O^(t)S_i,k_*) and those who die (e.g., *E_i,k_^O^(t)(1-S_i,k_*)), for each SES group; *S_i,k_* is the survival fraction per year for each age group *i* and SES group *k* (unit: % per year). The parameters *τ_1,i,k_* and *τ_2,i,k_* are the transference rates (TRs) that correspond to the fraction of individuals per year from the *not overweight* and the *overweight* categories that become *overweight* and *obese* for each age group *i* and SES group *k*, respectively (unit: % per year). The parameters *τ_3,i,k_* and *τ_4,i,k_* are the TRs of individuals from the *obese* to the *overweight* category and from the *overweight* to the *not overweight* category, respectively, for each age group *i* and SES group *k* (unit: % per year) (see the flow diagram in Fig 1 for a global view of the population aging structure). The case of *i* = 0, which corresponds to ages 0 to four years, is shown explicitly within the figure because of its slight difference from the other age groups.

The number of births per year for each BMI category and SES group was determined using the following equations:

$$B_{N,k}(t)=\mu\theta_{N,k}\left( \frac{f(t)}{\left( Y_{F}-Y_{I}+1 \right)} \right)\sum_{i=Y_{I}}^{Y_{F}} \left( N_{i,k}(t)+W_{i,k}(t)+O_{i,k}(t) \right) \left( 7 \right)$$

$$B_{W,k}(t)=\mu\theta_{W,k}\left( \frac{f(t)}{(Y_{F}-Y_{I}+1)} \right)\sum_{i=Y_{I}}^{Y_{F}} \left( N_{i,k}(t)+W_{i,k}(t)+O_{i,k}(t) \right) (8)$$

$$B_{O,k}(t)=\mu\theta_{O,k}\left( \frac{f(t)}{(Y_{F}-Y_{I}+1)} \right)\sum_{i=Y_{I}}^{Y_{F}} \left( N_{i,k}(t)+W_{i,k}(t)+O_{i,k}(t) \right) (9)$$

where *µ* is the total fraction of women in the population aged 15 to 49 years (we used the fraction of urban women in 2005 according to the ENDS survey, *µ=0.523*, unit: % per year); *μ∑_i=YI_^YF^((N_i,k_(t) + W_i,k_(t) + O_i,k_(t))* is the female population of childbearing age at time *t* for each age group *i* and SES group *k*, respectively (unit: women); *f* is the total number of children born from each woman during the childbearing years (fertility rate) at time *t* (births for each woman, unit: child per woman ); *θ_N,k_, θ_W,k_,* and *θ_O,k_* are the fractions of births by each BMI category and SES group *k* (unit: % per year) (assumed to be the prevalences by BMI category and SES group of children aged 0 to 2 months); and Y_I_ and Y_F_ (unit: year) are the first and last childbearing years considered, which we assumed to be 15 to 49, respectively.


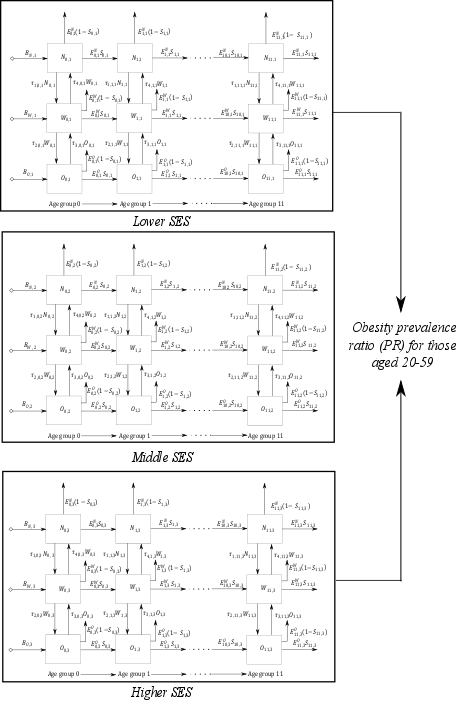


**Fig 1. Overview of the SD model structure by SES**

The exit rates per year by BMI category, age, and SES group were modelled using the following equations:

$$E_{0,k}^{N}\left( t \right)=\left( \frac{B_{N,k}\left( t \right)+{\tau_{4,0,k}W}_{0,k}\left( t \right)-{\tau_{1,0,k}N}_{0,k}\left( t \right)}{Y} \right) (10)$$

$$E_{0,k}^{W}(t)=\left( \frac{B_{W,k}\left( t \right)+{\tau_{1,0,k}N}_{0,k}\left( t \right)+{\tau_{3,0,k}O}_{0,k}\left( t \right)-W_{0,k}(t)\left( \tau_{2,0,k}+\tau_{4,0,k} \right)}{Y} \right) (11)$$

$$E_{0,k}^{O}(t)=\left( \frac{B_{O,k}(t)+{\tau_{2,0,k}W}_{0,k}(t)-{\tau_{3,0,k}O}_{0,k}(t)}{Y} \right) (12)$$

$$E_{i,k}^{N}\left( t \right)=\left( \frac{E_{i-1,k}^{N}{\left( t \right)S}_{i-1,k}+{\tau_{4,i,k}W}_{i,k}\left( t \right)-{\tau_{1,i,k}N}_{i,k}\left( t \right)}{Y} \right), for i\in\left( 1,\ldots,n-1 \right) \left( 13 \right)$$

$$E_{i,k}^{W}\left( t \right)=\left( \frac{E_{i-1,k}^{W}\left( t \right)S_{i-1,k}+{\tau_{1,i,k}N}_{i,k}\left( t \right)+{\tau_{3,i,k}O}_{i,k}\left( t \right)-W_{i,k}\left( t \right)\left( \tau_{2,i,k}+\tau_{4,i,k} \right)}{Y} \right), for i\in\left( 1,\ldots,n-1 \right) (14)$$

$$E_{i,k}^{O}\left( t \right)=\left( \frac{E_{i-1,k}^{O}\left( t \right)S_{i-1,k}+{\tau_{2,i,k}W}_{i,k}\left( t \right)-{\tau_{3,i,k}O}_{i,k}\left( t \right)}{Y} \right), for i\in\left( 1,\ldots,n-1 \right) (15)$$

where *Y = 5 years,* corresponding to the average time individuals spend in a given age group before maturing into the next age group. The equations (10-12) correspond to the exit rates per year by BMI category and SES group for the age group 0 to four years. Finally, the survival fraction for each age group is determined by:

$$S_{i}=\exp\left( R_{i}Y \right), for i\in\left( 0,\ldots,n-1 \right) (16)$$

In this formulation, $R_{i}$ is the mortality rate per year for each age group *i* (unit: % per year). We assumed the same mortality rate for each BMI category and SES group in each age group *i*, but differed across age groups.

The data processing was conducted using SAS 9.3 (SAS Institute Inc.) and Mathematica 9.0.1 (Wolfram Research, Inc.), and all simulations were run on iThink 9.0.2 (ISEE Systems, Inc.).

**Section 2. Heuristic to estimate the transference rates between BMI categories by age and SES**

We used the proposed heuristic for estimating the TRs (parameters) between BMI categories by age and SES groups using data from the ENDS of the 2005 and 2010 (historical data). We initialized the heuristic using the distribution of BMI categories by SES from 2005 ENDS. For the purposes of this SD model, we assumed that the TRs are uniform within each five-year age group, but differed across age groups.

To calculate the TRs by BMI category, SES, and age group we employed the following heuristic. First, we estimate the prevalence rates by BMI category and age *j* in 2010 for each age group *i* and SES group *k* using the below system of equations:

$$P_{i+1,j,k}^{'}=B_{i,k}.P_{i,j,k} (17)$$

where

$B_{i,k}=A_{i,k}.(A_{i,k}.\left( A_{i,k}.\left( A_{i,k}.A_{i,k} \right) \right))$;

$A_{i,k}=\left( \begin{matrix} \alpha_{1,i,k}-\tau_{1,i,k} & \tau_{4,i,k} & 0 \\ \tau_{1,i,k} & \alpha_{2,i,k}-\tau_{2,i,k}-\tau_{4,i,k} & \tau_{3,i,k} \\ 0 & \tau_{2,i,k} & \alpha_{3,i,k}-\tau_{3,i,k} \end{matrix} \right)$; $P_{i+1,j,k}^{'}=\left( \begin{matrix} p_{N10, i+1,j,k}^{'} \\ p_{W10,i+1,j,k}^{'} \\ p_{O10,i+1,j,k}^{'} \end{matrix} \right)$;$P_{i,j,k0}=\left( \begin{matrix} P_{N05,i,j,k} \\ P_{W05,i,j,k} \\ P_{O05,i,j,k} \end{matrix} \right)$;

where *j* ∈ (1,….,5) is the corresponding year in each age group *i*; *i* ∈ (0,….,11) represents intervals of five years (0–4, 5–9,...,55–59, and 60–64); *k* ∈ (1, 2, 3) represents the SES groups (lower, middle, and higher SES); *P_N05,i,j,k_*, *P_W05,i,j,k_*, and *P_O05,i,,j,k_* are the prevalence rates by BMI category and age *j* in 2005 for age group *i* and SES group *k*; *α_1,i,k_, α_2,i,k_,* and *α_3,i,k_* are the retention rates for individuals by BMI category, age group *i,* and SES group *k*, corresponding to the fraction of individuals who remain in the same BMI category between 2005 and 2010; *τ_1,i,k_*, *τ_2,i,k_* *τ_3,i,k_*, and *τ_4,I,k_* are the TRs that are used to run the proposed SD model by SES; and *P’_N10,i+1,,j,k_*, *P’_W10,i+1,j,k_*, and *P’_O10,i+1,,j,k_* are the estimated prevalence rates by BMI category and age *j* in 2010 for age group *i* and SES group *k* five years later. The matrix *A_i,k_* represents the equations used to calculate the estimated prevalence rates by BMI category in the year *t+1* for age group *i* and SES group *k,* where *t* is 2005. To estimate the prevalence rates by BMI category, age, and SES group in the year 2010 (*t+5*), the heuristic considers changes in the prevalence rates by BMI category for each year as a function of the prevalence rates by BMI categories obtained from 2005 ENDS. This heuristic therefore, estimates the TRs aggregated over five years.

After estimating the prevalence rates by BMI category, age, and SES group in 2010, the heuristic calculates the TRs by BMI category, age and SES group using a minimization process. This process involves minimizing the difference between prevalence rates by BMI categories, age, and SES groups reported in the 2010 ENDS and the estimated 2010 prevalence rates informed by data from the 2005 ENDS. Specifically, the TRs were estimated by solving the system of equations (17) for each age group *i* and SES group *k,* and minimizing the following equation using the FindMinimum function in Mathematica 9 [3]:

$${Min QD}_{i,k}=\sum_{j=1}^{5} \left[ \left( P_{N10,i+1,j,k}-p_{N10,i+1,j,k}^{'} \right)^{2}+\left( P_{W10,i+1,j,k}-p_{W10,i+1,j,k}^{'} \right)^{2}+\left( P_{O10,i+1,j,k}-p_{O10,i+1,j,k}^{'} \right)^{2} \right], (18)$$

with the restrictions

$$\alpha_{1,i,k}+\tau_{1,i,k}=1$$

$$\alpha_{2,i,k}+\tau_{2,i,k}+\tau_{4,i,k}=1$$

$\alpha_{3,i,k}+\tau_{3,i,k}=1$ (19)

$$0\leq\alpha_{l,i,k}\leq1, (l=1, 2, 3)$$

$$0\leq\tau_{m,i,k}\leq1, \left( m=1, 2, 3, 4 \right)$$

where *P_N10,i+1,j,k_*, *P_W10,i+1,j,k_*, and *P_O10,i+1,j,k_* are the prevalence rates by BMI category and age *j* in 2010 for age group *i* and SES group *k*. The FindMinimum function searches for a local minimum in a function including several variables and constraints.

**References**

1. Rutstein SO, Jonhson K. The DHS Wealth Index. DHS Comparative Reports no. 6. Calverton, MA: ORC Macro; 2004.

2. Sterman JD. Business Dynamics: Systems Thinking and Modeling for a Complex World. USA: McGraw-Hill/Irwin; 2000.

3. Meisel JD, Sarmiento OL, Olaya C, Valdivia JA, Zarama R. A system dynamics model of the nutritional stages of the Colombian population. Kybernetics. 2016;45: 554–570. doi:http://dx.doi.org/10.1108/K-01-2015-0010

4. Departamento Administrativo Nacional de Estadística. Series de población [Internet]. [cited 11 Jul 2017]. Available: http://www.dane.gov.co/index.php/estadisticas-por-tema/demografia-y-poblacion/series-de-poblacion

5. World Health Organization. WHO Child Growth Standards: Length/height-for-age, weight-for-age, weight-for-length, weight-for-height and body mass index-for-age: Methods and development [Internet]. Geneva; 2006. Available: http://www.who.int/childgrowth/standards/technical_report/en/index.html

6. de Onis M, Onyango AW, Borghi E, Siyam A, Nishida C, Siekmann J. Development of a WHO growth reference for school-aged children and adolescents. Bull World Health Organ. 2007;85: 660–667.

7. Clinical guidelines on the identification, evaluation, and treatment of overweight and obesity in adults: executive summary. Expert Panel on the Identification, Evaluation, and Treatment of Overweight in Adults. Am J Clin Nutr. 1998;68: 899–917.
